# Maintenance of Barrier Tissue Integrity by Unconventional Lymphocytes

**DOI:** 10.3389/fimmu.2021.670471

**Published:** 2021-04-14

**Authors:** Joshua R. Cox, Sheena M. Cruickshank, Amy E. Saunders

**Affiliations:** ^1^ Manchester Collaborative Centre for Inflammation Research, Division of Infection, Immunity and Respiratory Medicine, School of Biological Science, Faculty of Biology, Medicine and Health, University of Manchester, Manchester Academic Health Science Centre, Manchester, United Kingdom; ^2^ Lydia Becker Institute of Immunology and Inflammation, Division of Infection, Immunity and Respiratory Medicine, School of Biological Science, Faculty of Biology, Medicine and Health, University of Manchester, Manchester Academic Health Science Centre, Manchester, United Kingdom

**Keywords:** innate lymphoid cell, mucosal-associated invariant T cell, γδ T cell, repair, barrier, review

## Abstract

Mucosal surfaces, as a first barrier with the environment are especially susceptible to damage from both pathogens and physical trauma. Thus, these sites require tightly regulated repair programs to maintain barrier function in the face of such insults. Barrier sites are also enriched for unconventional lymphocytes, which lack rearranged antigen receptors or express only a limited range of such receptors, such as ILCs (Innate Lymphoid Cells), γδ T Cells and MAIT (Mucosal-Associated Invariant T Cells). Recent studies have uncovered critical roles for unconventional lymphocytes in regulating mucosal barrier function, and, in particular, have highlighted their important involvement in barrier repair. The production of growth factors such as amphiregulin by ILC2, and fibroblast growth factors by γδ T cells have been shown to promote tissue repair at multiple barrier sites. Additionally, MAIT cells have been shown to exhibit pro-repair phenotypes and demonstrate microbiota-dependent promotion of murine skin healing. In this review we will discuss how immune responses at mucosal sites are controlled by unconventional lymphocytes and the ways in which these cells promote tissue repair to maintain barrier integrity in the skin, gut and lungs.

## Introduction

Mucosal and barrier sites such as the skin, lungs and digestive tract are major contact points with the external environment. As such, they are at risk from damage by infection, environmental toxins and physical trauma. Mucosal and barrier sites have therefore evolved features to withstand such insults, with some sites particularly specialized in dealing with continued damage. These external-facing sites also have the added challenge of being colonized by diverse commensal microbial species, whose cooperative co-existence with their host is often vitally dependent upon appropriate compartmentalization. Commensal-immune system interactions can promote and enhance barrier function for example enhancing normal cell turnover and barrier integrity. Tissue injury, however, represents a particular challenge at mucosal and barrier sites, as damage will inevitably lead to a failure of microbial compartmentalization and thus necessitate an anti-microbial response. The coordination of immune responses that can both work with commensal microbes to promote barrier function in everyday “wear and tear” as well as recognize and repair damage is a major challenge. Dysregulation of microbial interactions at the barrier and defective repair can lead to disease or worsen prognosis in chronic skin wounds in diabetic and elderly individuals, inflammatory bowel disease (IBD) and COPD (Chronic obstructive pulmonary disease) (1). Data are emerging of a key role for unconventional lymphocytes in helping regulate these critical barrier functions.

Mucosal barriers are particularly enriched for ‘unconventional lymphocytes’, defined as lymphocytes either lacking rearranged antigen receptors, or expressing antigen receptors with a limited repertoire-innate lymphoid cells, mucosal-associated invariant T cells (MAIT) and γδ T cells. Innate lymphoid cells (ILC) do not express functional T cell receptor (TCR) proteins whereas, MAIT possess a semi-invariant TCR restricted for the non-classical MHC class I molecule, MR1 (2). γδ T cells can also be considered an unconventional lymphocyte as during development, monoclonal or oligoclonal populations develop, expressing semi-invariant TCR with effector fates also imprinted at this stage (3). γδ T cells have been described as ‘adaptate’ since these cells display features of both innate and adaptive systems (4). Based on our definition of unconventional lymphocytes NK and iNKT cells could also be included, however in this review we will focus on lymphocytes with compelling evidence for a role in repair, namely the ILCs, γδ T cells and MAIT.

Recent work has highlighted that unconventional lymphocytes possess a shared trait, as important orchestrators of repair at mucosal and barrier tissues, which sets them apart from most conventional lymphocytes. Unconventional lymphocytes share the ability to rapidly respond to damage and cytokine cues *in situ* and possess many pro-repair strategies that will be discussed here.

## Innate Lymphoid Cells (ILC)

ILC encompass NK cells, LTi (Lymphoid tissue inducer) cells and helper ILCs (ILC1, ILC2 and ILC3), which are a relatively recently discovered cell class and our understanding of their function and ontology is growing at a rapid pace. Helper ILC exclude NK cells since these differentiate prior to the common helper ILC progenitor (CHILP) (5) and possess more cytotoxic functions while helper ILC display functions more similar to T-helper cells. LTi cells are also distinct from helper ILCs as although they are part of the ILC3 family, they follow a separate developmental program and are only present in a developmental window before birth, where they are crucial for the development of secondary lymphoid organs (6).

ILC are grouped in a similar way to their T-helper counterparts, with each subset possessing discrete functions and phenotypes. ILC1 are analogous to Th1 and provide the type-1 immunity arm of ILC. ILC1 require the transcription factor Tbet for functionality (7), they primarily produce IFNγ, particularly in response to IL-12 and they provide an important early response to viral infections (8). While there were initial difficulties distinguishing ILC1 from NK cells, it is now appreciated that ILC1 are less cytotoxic than NK cells and function in a ‘helper’ role (7).

ILC2 are analogous to Th2 cells, requiring GATA3 as their master transcription factor and producing IL-5, IL-13 and IL-9 (9), although murine ILC are not generally efficient producers of IL-4 (10). ILC2 are regulated by a large range of tissue signals including prostaglandins, neuropeptides, metabolic cues and alarmins such as IL-25, IL-33 and IL-18 released by damaged epithelial cells (9). Their roles have largely been studied in responses to helminths, which generally induce extremely strong type-2 responses, in lung allergy models and in atopic conditions such as atopic dermatitis.

ILC3 are akin to Th17 cells requiring the transcription factor RORγt. They respond to IL-1β and IL-23 by producing the type-17 mediators IL-17A, IL-17F and IL-22 (6). Prior to birth, LTi cells which are part of the ILC3 family, are one of the earliest immune cell types to populate the intestine where they play a crucial role in secondary lymphoid organogenesis. In adults three distinct populations of ILC3 exist, defined by expression or absence of CCR6 and NK cell receptors (NCR), NKp46 (mice) or NKp44 (humans) (6, 11). NCR^+^ ILC3 share transcriptional overlap with ILC1 and possess functional plasticity to take on an ILC1-like phenotype (11). NCR^-^ CCR6^+^ LTi-like cells share a cell surface phenotype similar to fetal LTi cells, but follow a developmental program common to ILC1, 2 and 3 which is distinct from that of LTi cells (6). NCR^-^ CCR6^-^ ILC3s have also been identified in the intestine where they are thought to be precursor cells for the NCR^+^ ILC3s (6).

### ILC1

**Figure 1:**
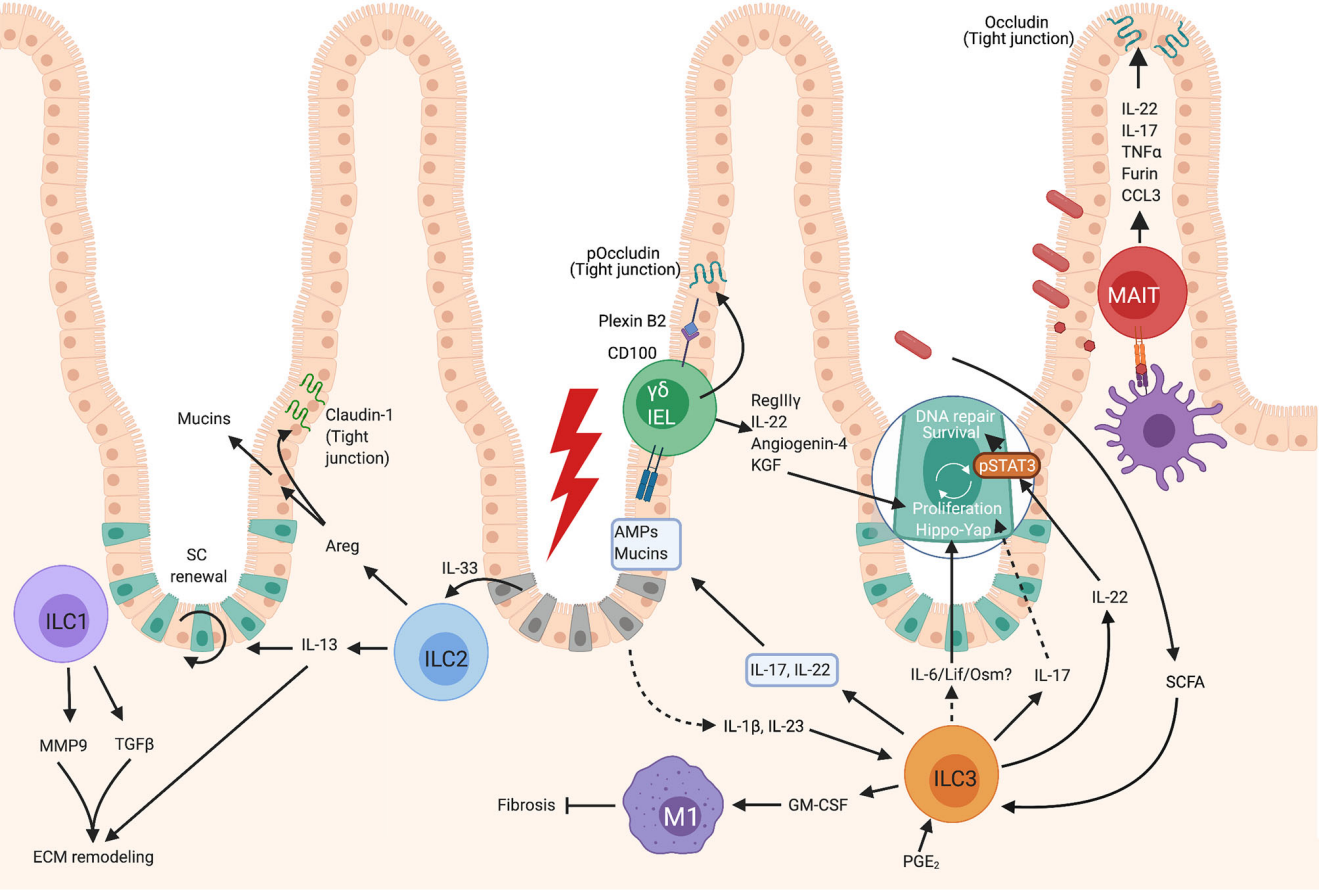
Roles of unconventional lymphocytes in intestinal repair. Gut ILC1 can produce MMP9 and TGFβ1 which contribute to extracellular matrix remodeling- a crucial part of repair. ILC2s become activated after exposure to IL-33 released by damaged epithelium, which results in their production of IL-13, which contributes to extracellular matrix remodeling and epithelial cell proliferation. Activated intestinal ILC2 also produce Areg which both enhances mucin production by epithelial cells increasing the mucus layer, and upregulates Claudin-1, increasing tight junction strength and reinforcing the barrier. Gut γδ T cells, or γδ IELs, can contribute to repair by the production of KGF which promotes epithelial proliferation, and *via* the production of AMPs such as RegIIIγ. In gut, the contribution of MAIT cells to healing responses remains relatively unknown, however these cells have been observed to respond similarly to human peripheral blood MAIT and can strengthen epithelial barrier integrity by inducing the tight junction protein, occludin, and by enhancing mucin expression to bolster the mucus layer. Intestinal ILC3s can become activated by PGE_2_, SCFAs, or IL-23 and IL-1β. Activated ILC3s produce IL-22 which promotes epithelial stem cell maintenance in a STAT3 dependent manner, and can also promote the DNA damage response. ILC3s can also promote epithelial cell proliferation *via* a Hippo/Yap-1 pathway downstream of gp130. The factors released by ILC3s which signal *via* gp130 are not known, but may be IL-6, Lif or Oncostatin M as these all signal *via* gp130-coupled receptors. Activated ILC3s also produce GM-CSF which contributes to M1 polarization of macrophages which inhibit healing, but also inhibit fibrosis which is associated with scar formation. ILC3s can produce IL-22 and IL-17 which promotes the production of AMPs and mucins by epithelial cells, but it is not known if these cells are the key producers of these cytokines in this context. Created with BioRender.com.

The majority of studies describe pathological roles for ILC1 and protective roles have been less well characterized at barrier sites. Indeed, deviation from ILC3 phenotypes into ILC1-like cells has been described in IBD (12). ILC1 are rare within the healthy small intestine, but this subset becomes enriched in IBD (12) leading to the assumption that these cells contribute to pathology. However, recent work in murine and human intestinal organoids has demonstrated roles for gut ILC1 in promoting repair-like activities (13). In organoids, ILC1 can produce TGFβ1, resulting in both crypt bud formation and remodeling of the extracellular matrix. Furthermore, ILC1 produced MMP9, which degrades matrix components, causing stiffening of organoid-proximal ECM and degradation in distal regions. As ECM remodeling is an integral part of healing, this mechanism may contribute to repair (**Figure 1**).

While there is limited literature on skin ILC1, these cells are increased in injured murine skin during the proliferative phase of healing (14). It is therefore likely that they contribute to skin healing in a comparable way to gut, involving TGFβ production and matrix remodeling. Indeed, in cutaneous healing TGFβ is known to be elevated during the proliferative phase (15) correlating with ILC1 numbers (14). Thus, there is the potential for ILC1 to contribute to healing by regulating matrix remodeling and cell proliferation *via* production of TGFβ, however, evidence of a clear role for ILC1 in repair is currently lacking.

### ILC2

Much of ILC2 biology at mucosal sites has been investigated in the context of allergic or anti-helminth responses, where type-2 responses are essential. Since invasion of multicellular parasites causes a large amount of tissue damage, it is, perhaps, unsurprising that ILC2 also promote epithelial repair and barrier integrity.

**Figure 2:**
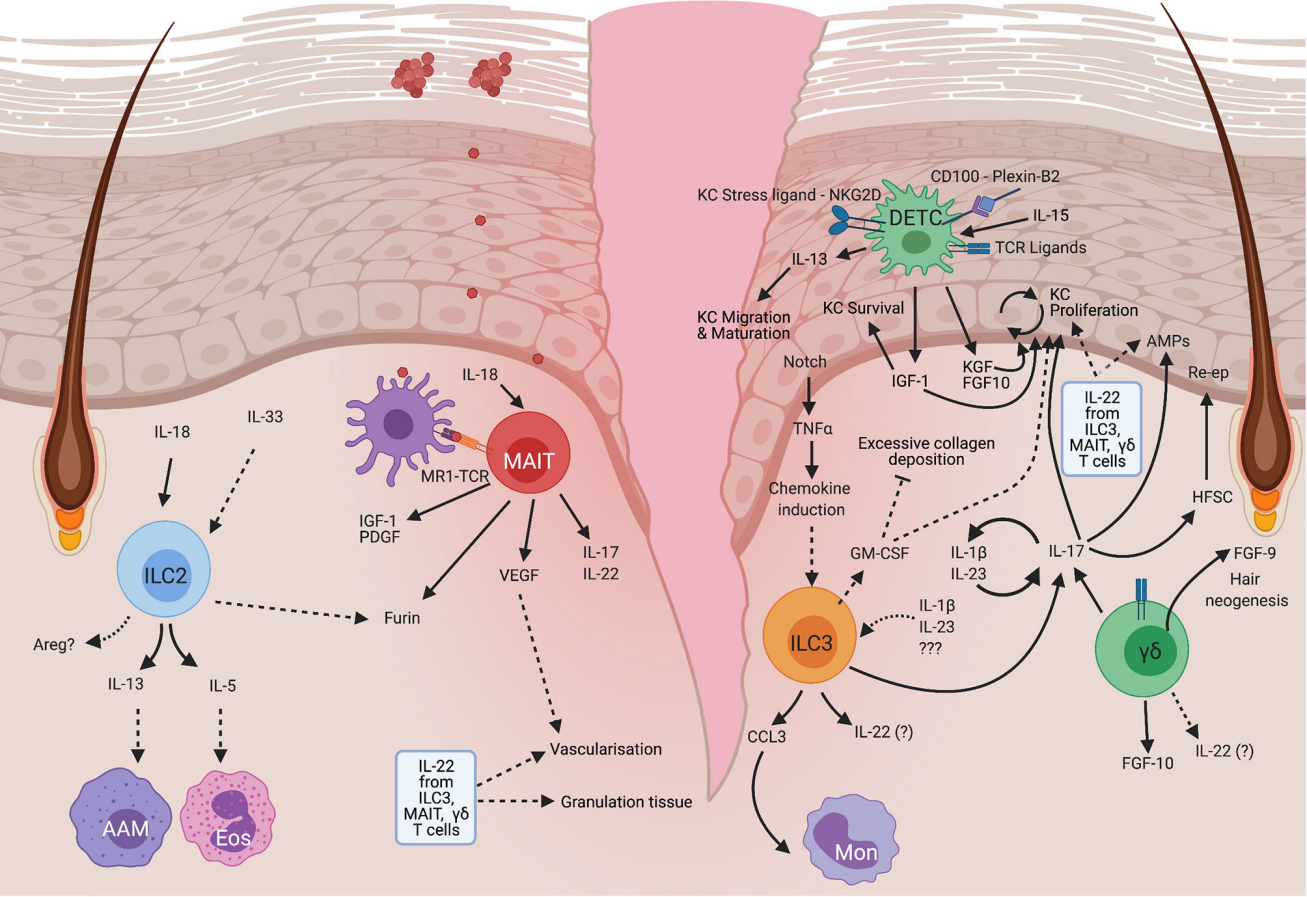
Mechanisms of unconventional lymphocytes in mouse skin repair. ILC2 become activated in response to IL-18 released by damaged cells, resulting in the production of IL-13 which can drive the alternative activation of macrophages (AAM), IL-5 which recruits eosinophils (Eos), and potentially Areg which has been shown to have many pro-repair roles at other barrier sites but is yet to have a role defined in skin. MAIT cells become activated by TCR engagement and IL-18 which results in PDGF and IGF-1 production that enhances keratinocyte survival. These cells are also thought to modulate angiogenesis which is an important process for tissue remodeling in repair. MAIT cells can also produce IL-17, but a direct role for MAIT-derived IL-17 in skin repair has not yet been demonstrated. DETC are a mouse specific cell type that is maintained by IL-15 produced by keratinocytes. On damage DETC become activated in response to TCR signals, CD100 engagement with plexin B2 on epithelial cells and detection of stress ligands *via* NKG2D. This stimulates DETC to produce IGF1 which promotes keratinocyte survival. IGF1, alongside DETC-derived KGF and FGF10 also promote epidermal proliferation. DETC can produce IL-13, which in skin, regulates keratinocyte maturation and migration, but may also have other roles in common with that seen at other barrier sites. ILC3 can become activated downstream of Notch-dependent TNFα production by keratinocytes, or by IL-23 and IL-1β. Activated ILC3 produce CCL3 which recruits monocytes (Mon). ILC3 may also produce GM-CSF which promotes reepithelialisation and vascularization, and regulates collagen deposition. ILC3 are particularly well known for their ability to produce IL-17A which at low levels is pro-repair and acts by promoting the production of antimicrobial peptides and stimulating keratinocyte proliferation. ILCs can also produce IL-22 and IL-17F which upregulates AMPs. IL-22 which can also enhance the formation of granulation tissue, and stimulate vascularization. Similar to ILC3, dermal γδ T cells, which are mainly Vγ4^+^, produce IL-17 and can form a positive feedback loop by stimulating IL-23 and IL-1β production, leading to enhanced activation of both ILC3 and dermal γδ T cells, which is proposed to impair healing. These dermal γδ T cells can also produce FGF10 which promotes keratinocyte proliferation and FGF9 which stimulates hair follicle neogenesis and is thus associated with regeneration. HFSC, Hair follicle stem cells, Re-ep, Re-epithelialization. Created with BioRender.com.

ILC2 have been shown to accumulate post skin wounding during either the inflammatory phase in mice (16), or the later proliferative or remodeling phases in mice (14) and humans (16). This expansion of ILC2 in response to tissue injury implies that they are involved in the repair process (**Figure 2**) however, clarification of the timing of ILC2 accumulation may provide additional insight into their specific roles in healing.

**Figure 3:**
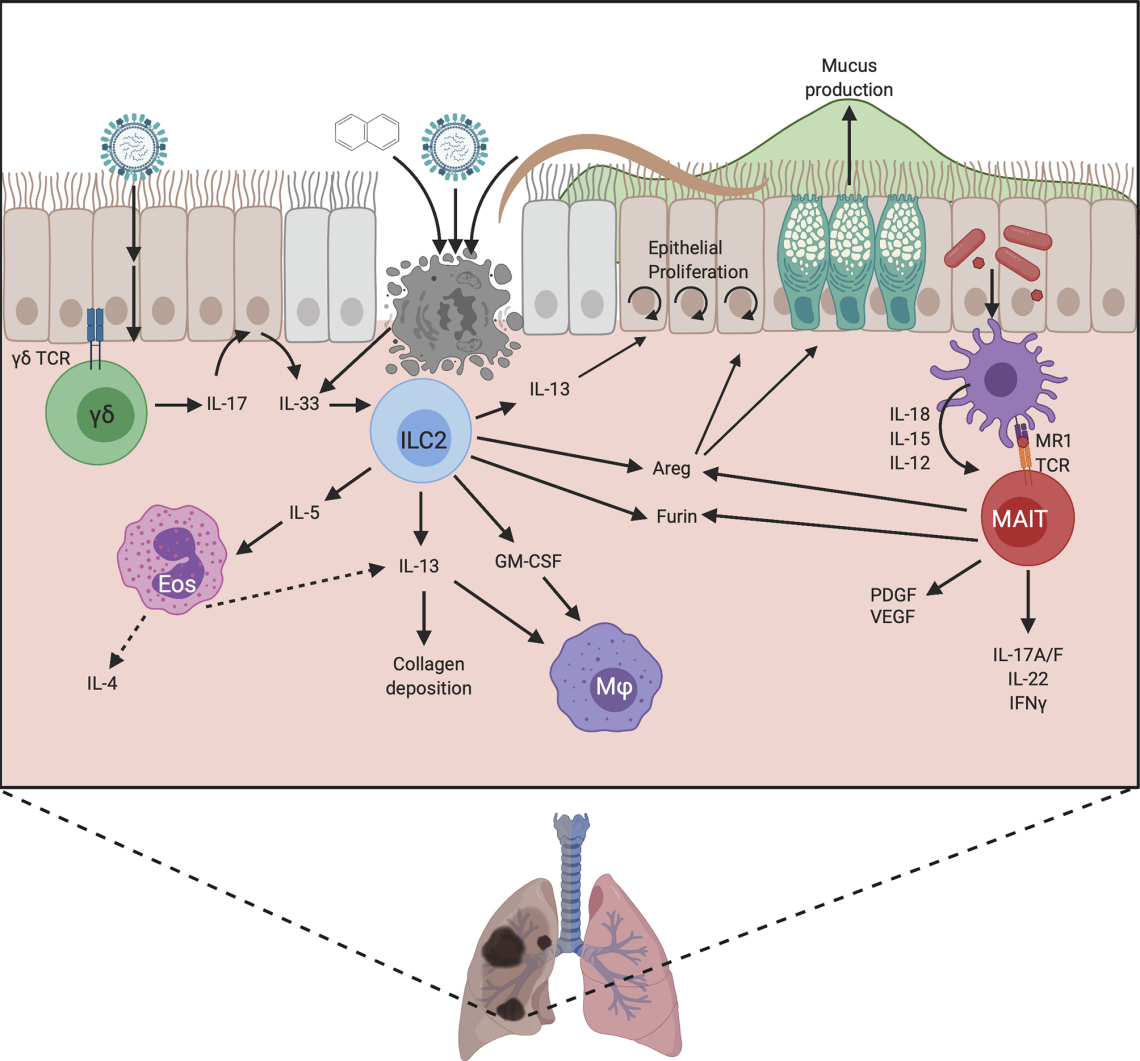
Proposed mechanisms by which unconventional lymphocytes promote lung repair. Damage can be induced by chemical exposure or infection with viruses or helminths, which leads to IL-33 release by damaged epithelium. This activates ILC2, stimulating the production of cytokines and Areg. Areg promotes epithelial cell proliferation, reepithelialisation, and goblet cell activity, strengthening the mucus barrier. The production of IL-13 and GM-CSF by ILC2 recruits macrophages and promotes their alternative activation, allowing them to contribute to repair. IL-13 also promotes epithelial cell differentiation and proliferation, and modulates collagen deposition. ILC2s also produce IL-5 which recruits eosinophils. These eosinophils can produce IL-4 and IL-13 and may therefore also contribute to healing. ILC2s may contribute to repair by upregulating Furin to promote the activation of MMPs and TGFβ, and they may directly regulate the extracellular matrix, but these mechanisms are yet to be proven. In neonatal lung γδ T cells respond to infection by producing IL-17 which stimulates IL-33 release by epithelial cells. Lung MAIT cells can become activated by TCR ligands from microbes, and cytokine signals from damaged cells, leading to the production of IFNγ, IL-17A/F and IL-22 which can promote epithelial proliferation. MAIT cells can also produce growth factors such as PDGF and VEGF which play a role in repair. Similar to ILC2s, MAIT cells can produce Areg which promotes epithelial cell turnover and goblet cell activity, and they may also upregulate furin to increase protein cleavage and activation. Created with BioRender.com.

One role for ILC2 is in modulating epithelial activity. On tissue damage, IL-33 is released by necrotic cells which promotes ILC2 accumulation as seen in the lungs of influenza-infected mice (17). These ILC2 regulate epithelial cell proliferation and necrosis, and promote goblet cell activity (17) thus maintaining epithelial integrity and protecting from severe pathology (**Figure 3**).

Much of the ILC2 data to date has used helminth infection models. However, the specific pro-repair roles of ILC2 are challenging to disentangle since ILC2 promote worm expulsion (18) as well as having potential repair roles. However, in later stages of helminth models once worm expulsion/killing has been achieved, an examination of reparative processes can occur. For example, in *Nippostrongylus brasiliensis* infection, ILC depletion results in emphysema-like pathology, microbleeding and impaired lung capacity (19), demonstrating a crucial repair role for ILCs. The cells responsible for repair in this model are ILC2 producing amphiregulin, IL-9, IL-13 and IL-5 and they also promote eosinophil recruitment and the alternative activation of macrophages (19) (**Figure 3**).

Production or modulation of extracellular matrix proteins by ILC is a relatively unstudied, but potentially important repair mechanism. For instance dermatopontin, decorin and asporin are highly expressed by lung ILC (17). Although these make up a significant proportion of ‘wound healing’ gene signatures, the role of extracellular matrix components in healing remains under-explored, and the relative contribution of ILC2s to the production of these also remains unknown.

Therefore, the best studied contributions of ILC2 to repair are *via* the production of cytokines or growth factors, and the contribution of these ILC2 products to the repair process will be discussed further here.

#### IL-13 and IL-5

ILC2 react quickly to injury-induced IL-33 by producing IL-13 and IL-5 (16, 20) (**Figures 1**–**3**). There is variability in the responses at different tissue sites and skin ILC2 display reduced IL-33 responsiveness compared with ILC2 at other sites (21). In the skin, the IL-1 family alarmin cytokine, IL-18, appears to play a more dominant role in the activation of skin ILC2 (21). Regardless of the alarmin required for stimulation, IL-13 production by ILC2 is one of the key outcomes. This cytokine has established links to tissue repair by promoting pro-repair phenotypes of macrophages, collagen deposition, and the proliferation and differentiation of epithelial cells (22).

In intestinal damage models IL-13 signaling is crucial for epithelial regeneration by promoting LGR5^+^ crypt stem cell self-renewal (23) (**Figure 1**). A defect in LGR5^+^ cell number was also seen in ILC deficient mice at steady state, and this was ameliorated by transfer of ILC2 which subsequently localized closely around intestinal crypts (23). This defect in LGR5^+^ cell numbers was not restored by transfer of IL-13-deficient ILC2, demonstrating that IL-13 production by ILC2 contributes to repair *via* regulating stem cells (23). While not a major finding in the study, Dahlgren et al. identified ILC2 localization predominantly around hair follicles within the skin (24). Hair follicles are an important stem cell niche which can contribute to healing of even the interfollicular epidermis (25–27), which could also suggest the involvement of a stem cell-ILC2 axis in skin repair.

Another suggested role for ILC2 in repair is *via* the promotion of macrophage alternative activation. A recent study investigating lung repair, in a naphthalene-induced bronchial damage model, identified a synergistic role for IL-13 and IL-33 in promoting the development of pro-healing alternatively activated macrophages (AAM) (20) (**Figure 3**). ILC2 were identified as the primary producers of IL-13 in this model and their presence was required for AAM development and recruitment of ST2^+^ macrophages. The absence of ILC resulted in a defect in regeneration that was rescued by ILC2 transfer (20). In addition, these ILC2 also produced GM-CSF which plays a key role in macrophage development (20). It is likely that the influence of ILC2 on myeloid cells, in particular macrophages but potentially also eosinophils (via IL-5), is important for repair (**Figures 2** and **3**), especially as ILC2 are a major, innate source of IL-13 which can be produced more rapidly than adaptive sources, particularly in sterile injury. Therefore, the production of IL-13 is likely a key mechanism by which ILC2 contribute to repair by promoting stem cell maintenance and the alternative activation of macrophages.

ILC2 also produce IL-5 in response to activation, which does not yet have clear links to healing and may instead act to recruit eosinophils, which do not currently have a clearly defined role in repair but have been suggested to play a role in barrier integrity (28). Furthermore, eosinophils themselves are a major source of cytokines such as IL-4 and IL-13 that can in turn promote the generation of pro-repair macrophages (29, 30) thus ILC2 may be acting as a critical first line in aiding and coordinating the recruitment of other pro-repair cell types.

#### Amphiregulin

In addition to production of IL-13, ILC2s are also a major source of EGFR ligand amphiregulin (Areg) (**Figure 3**). Areg has well-established roles as a growth factor promoting tissue repair and having immunomodulatory activities (31). Indeed, in the context of influenza-induced damage, either lung ILC transfer or amphiregulin administration restores epithelial integrity in the absence of ILCs (17). A similar activity is also seen in the DSS colitis model where IL-33 is induced in response to epithelial damage. IL-33 promotes ILC2 Areg production which in-turn enhances epithelial claudin-1 and mucin production to enhance tight barrier function and mucus generation (32) (**Figure 1**). Similar observations were made using an Itk-deficient mouse model. Itk-deficient mice have greatly reduced numbers of lamina propria ILC2 and fail to repair DSS induced intestinal damage resulting in enhanced mortality (33). It would be interesting to understand if this IL-33/Areg axis also plays a role in skin healing by promoting epithelial tight junction integrity. Transcriptome data suggest Areg is expressed by both murine (21, 34) and human skin ILC2, (**Figure 2**) with increased Areg expression observed in atopic dermatitis, perhaps reflecting attempts to repair the barrier defects associated with this condition (35). This reinforces the possibility that ILC2 production of Areg is a common pro-repair mechanism employed by ILC2 at multiple barrier sites, but further research is needed to show this.

#### Furin

ILC are a source of the endoproteinase furin. Furin activates a wide range of pro-protein substrates in the secretory pathway of all cell types. Furin is increased in ILCs present during time points when healing occurs in *N. brasiliensis* infection (36). However, direct data to show ILC2 derived furin is reparative, is lacking. The pro-repair function of furin appears to be largely inferred from its role as a proprotein convertase, activating MMPs and TGFβ, and promoting epithelial to mesenchymal transition (EMT) that promotes healing (37, 38). Interestingly ILC2 from other sites express furin mRNA at steady state particularly skin ILC2 (21) (**Figure 2**). One possibility is that ILC2 production of furin promotes formation of the stratum corneum by processing filaggrin (39). This could, potentially, also implicate furin as a pro-healing factor as it may enable the correct differentiation of the new epidermis in cutaneous wounding.

#### Heterogeneity in ILC2 Repair Function

There is increasing understanding of the significant heterogeneity among ILC2s and it is currently unclear if all ILC2 subpopulations promote repair to the same degree. For example, neonatal mouse lungs contain two defined subsets of mature, effector ILC2 (40). On stimulation with IL-33 and IL-7 the KLRG1^+^ ICOS^-^ ILC2 subset expresses high levels of IL-5 and IL-13, while KLRG1^-^ ICOS^+^ ILC2 express little IL-5 or IL-13 but produce large amounts of Areg (40). Therefore, in neonatal mouse lung there appears to be a proinflammatory KLRG1^+^ ILC2 subset and a pro-repair ICOS^+^ ILC2 subset (40). In adult mice this distinction between subsets is less clear, and on papain challenge, expression of ICOS and KLRG1 are both increased on lung ILC2. ICOS^+^ ILC2s do express a relatively ‘pro-repair’ gene expression profile (40) however, the relevance of many of these genes to repair in the context of ILCs, rather than epithelial cells, is unknown. Intriguingly, in both steady-state neonatal, and papain treated adult lungs, the presence of KLRG1^+^ ILC2 was reduced in IL-33 KO animals, while ICOS^+^ ILC2 were unaffected (40), suggesting the putative ‘pro-repair’ ILC2 have less reliance on IL-33 for their expansion.

In *N. brassiliensis* infection multiple clusters of lung ILC2 are present during the repair phase, and express high levels of Areg (36) (**Figure 3**). Single cell RNAseq was performed on these cells and analysis based on expression similarity allowed the construction of predicted lineage path trajectories. This analysis suggests that the ILC2 present in repair can develop from ILC2 already present in the infected tissue indicative perhaps of the role of the environment in shaping ILC2 phenotype and function (36). Alternatively, this observation may show that pro-repair ILC2 are a more specialized and differentiated subset than other ILC2s.

Overall, it is clear that ILC2s contribute to repair at many barrier sites both directly and indirectly *via* influencing the cellular microenvironment and potentially *via* ECM remodeling, however the relative contribution of each of these mechanisms to repair remains incompletely understood.

### ILC3

The type-17 immune response is vital to mucosal defense, particularly in the gut. Not only are these responses essential for defense against microbial threats, but also in restoration of epithelial barriers. ILC3, as key innate type-17 cells, have therefore also been shown to have critical roles in the response to damage of epithelium and repair of the barrier

The bulk of work investigating ILC3 in repair has focused on gut models. However, observations that ILC3 are rapidly recruited to skin wound margins in a manner dependent on epidermal Notch-driven TNFα expression, suggest they may play similar roles in other tissues (14) (**Figure 2**). The critical role of ILC3 in intestinal repair has been demonstrated using anti-Thy1 treatment of mice to deplete ILC and analyze the response to chemical induced gut inflammation using methotrexate. Although anti-Thy-1 depletes other lymphocytes in addition to ILCs, its use in RAG KO mice, which display normal healing, allows more specific ILC depletion, as T cells are absent (41). This approach suggested that ILC were indeed playing a key role (41). Both NCR^+^ and NCR^–^ ILC3 were found to be activated early post methotrexate treatment, resulting in IL-17, TNFα, IL-22 and GM-CSF production (41) (**Figure 1**). Indeed, when the role of ILC3 more specifically was examined using RORγt deficient animals, defects in crypt repair were observed, with an accompanying reduction in LGR5^+^ crypt stem cells, again demonstrating a critical role for ILC3 in promoting epithelial repair (41).

ILC3s can produce a range of inflammatory mediators, most notably IL-17, TNFα, IL-22 and GM-CSF, and it is thought that its these cytokines that are key to ILC3 repair function.

#### Co-Ordinating Mononuclear Phagocyte Activity

One way that ILC3 contribute to barrier repair is *via* coordinating immune cell activity in the wound. Skin wound ILC3 produce the chemokine CCL3 which is required for robust monocyte recruitment (**Figure 2**) in early healing, but is not required at later timepoints (14). Therefore, similar to ILC2, ILC3 have a role in coordinating myeloid cell activities which are important for tissue repair. In the intestine, however, ILC3-derived GM-CSF can promote antimicrobial M1-like macrophage development over typical type-2 ‘healing’ responses in macrophages (42) (**Figure 1**). ILC3-produced GM-CSF was suggested to temper reparative responses to avoid undesirable fibrosis. Indeed, in complicated human Crohn’s disease where strictures (narrowing and scarring of the intestine) occur, both epithelial to mesenchymal transition (EMT) genes, and macrophage signatures that are usually restrained by ILCs in colitis models, were increased (42). Depletion of ILCs showed that these cells limit collagen deposition pathways, epithelial to mesenchymal transition (EMT) and production of the pro-healing growth factor, platelet derived growth factor (PDGF) (42). However, contributions from all ILC subtypes cannot be excluded as all ILC types were depleted. The reduction in collagen deposition is likely due to ILC3-derived GM-CSF (42). Therefore, ILC3 act *via* production of GM-CSF which acts on monocytes and macrophages, to restrain some aspects of repair and avoid deleterious effects of scarring, ensuring an appropriate program of repair. GM-CSF can also have positive effects on skin repair, promoting re-epithelialisation, vascularization and controlling collagen deposition both directly, and by the induction of cytokines such as IL-6 (43, 44). ILC3 could potentially be a skin source of GM-CSF and thus may contribute to repair in this manner.

#### IL-22

ILC3 are a key source of IL-22, particularly in the intestine where they are the prominent producers (45) and the importance of this cytokine has been shown in several model systems. In a methotrexate-induced intestinal damage model the depletion of ILCs leads to impaired epithelial proliferation and reduced phosphorylation of STAT3. STAT3 phosphorylation is critical for intestinal repair which was shown to be partially dependent on IL-22 from ILC3, by the use of blocking antibodies (41) (**Figure 1**). However, a more recent paper employing IL-22 deficient mice demonstrated that IL-22 does not impact clinical score in methotrexate induced damage. Instead IL-22 helped to *maintain* the stem cell crypt rather than promoting epithelial proliferation (46). The proliferative response to intestinal damage instead requires ILC3-dependent gp130 signaling in crypt stem cells which activates the evolutionarily ancient Yap1-Hippo pathway (46). It is not fully understood how this pathway is activated in response to ILC3 but certainly at steady state ILC3 can produce Lif, Oncostatin M and IL-6 all of which signal *via* gp130-coupled receptors implicating gp130 signaling in regulating homeostasis of barriers (46) (**Figure 1**). Collectively this indicates at least 2 distinct mechanisms by which ILC3 promote intestinal repair with 1) an IL-22 and STAT3 dependent maintenance of stem cells (46) – likely *via* suppression of apoptosis (41) and 2) an IL-22-independent induction of Yap1-dependent proliferative responses and promotion of differentiation to pro-repair secretory cell types.

ILC3-derived IL-22 plays a role in protecting the gut epithelium under conditions of bacterial challenge. Systemic bacterial lipopolysaccharide (LPS) promotes prostaglandin E2 (PGE_2_) synthesis, which acts on ILC3 *via* the EP4 receptor to promote ILC3 proliferation, expression of signature genes such as *Rorc* and production of IL-22 (47) (**Figure 1**). Crucially, a lack of ILCs or ablation of EP4 expression in ILCs, results in systemic inflammation associated with bacterial translocation into the liver which is likely due to impaired gut barrier integrity. Similar to murine cells, human ILC3 also respond to PGE_2_ and this pathway is diminished in both neonates with sepsis, and in patients with other sepsis-related conditions (47) suggesting that ILC3 play a similar role in humans. This PGE_2_-IL-22 axis is also involved in responding to the damage incurred by DSS colitis (47) (**Figure 1**). Detection of the microbial metabolites, short chain fatty acids (SCFA), by ILC3 is important for IL-22 production and promoting epithelial barrier function as, in DSS colitis, ILC3 lacking Ffar2 – a receptor for SCFA, display reduced proliferation and impaired IL-22 production. This results in reduced production of mucins, and antimicrobial peptides such as Reg3α, β and γ, leading to worsened pathology (48).

Other studies have further emphasized the critical role of ILC3 in barrier repair and specifically the DNA damage response (DDR). Damage to the gut epithelium caused by the pro-carcinogen azoxymethane (AOM) or radiation induced damage repair both require IL-22 for the induction of DNA repair machinery and apoptotic responses to DNA damage (49) (**Figure 1**).

Overall the data point to an important role for ILC3-derived IL-22 in maintaining or restoring the intestinal barrier, but the importance of IL-22 production by ILC3s in repair at other sites is less well understood. The major source of gut IL-22 is ILC3 however, in skin γδ T cells are also an important source of IL-22 (50), and MAIT cells can also produce IL-22. Therefore, it is unclear if ILC3 are the key IL-22 producing cells in repair at all barrier sites, or if this is specific to the intestine.

In murine skin wound healing IL-22 is upregulated during the inflammatory phase (51). Interestingly, this response is blunted in diabetic mice that fail to heal in a timely fashion and the addition of recombinant IL-22 can restore healing by promoting keratinocyte proliferation (**Figure 2**), granulation tissue formation and vascularization (51). IL-22 can also improve healing of infected murine diabetic wounds which may be *via* its induction of host-defense and AMP gene expression (**Figure 2**), alongside promoting direct healing mediators such as Areg (52). The relative contribution of IL-22 produced by ILC3, γδ T cells and MAIT cells in skin remains incompletely understood, despite the importance of IL-22 in repair.

#### IL-17

IL-17 is a critical family of cytokines including both IL-17A and the less well studied IL-17F which are produced by ILC3, as well as γδ T cells and MAIT. ILC3 produce IL-17A, however IL-17F expression is the dominant wound response in skin (14) (**Figure 2**). The importance of ILC3 in skin healing was demonstrated by impaired healing in RORγt deficient animals that lack ILC3 and IL-17 production (14). This impeded re-epithelialisation, which was only restored by transfer of RAG KO splenocytes, which contain ILCs, but not T or B cells (14). While this does not definitively demonstrate an ILC3 role, the presence of ILC3 at the wound site post-transfer strongly suggests an ILC3 mediated effect.

While ILC3 roles have typically been split into epithelial maintenance/repair vs proinflammatory anti-microbial responses, the reality is likely much more nuanced. IL-17A can promote the production of anti-microbial peptides (AMPs) such as beta defensins, S100A8, lipocalin 2 and REG3A (mouse ortholog is RegIIIγ) from epithelial cells (53) (**Figures 1** and **2**). Indeed IL-17A and IL-17F have been reported to synergize with IL-22 in promoting AMP production (53). Both IL-17 and AMPs protect the barrier by controlling colonizing microbes and, promoting keratinocyte proliferation (54, 55). IL-17A may also affect keratinocyte proliferation by transactivating some configurations of EGFR receptors to influence stem cell activity (56), and by directing hair follicle stem cells to reconstitute the injured epidermis (56) (**Figure 2**). Indeed, in chronic diabetic skin wounds with a failure in re-epithelialization, there is evidence that type-17 pathways are underactive, at least at a transcriptional level (57), suggestive again of IL-17 promoting epithelial repair.

Although ILC3 and IL-17 play roles in repair, conventional and γδ T cells may be more potent sources of these cytokines, thus the relative contribution of ILC-derived IL-17 to repair remains unknown.

Collectively, these findings show that ILC3s have a role in repair, likely *via* their production of IL-22, IL-17 and GM-CSF and these cytokines may enable them to be involved in both epithelial regeneration responses, as well as limiting excessive reactions and scarring from occurring.

The contribution of other unconventional lymphocytes such as γδ T cells to IL-17 production will be discussed in the following section.

## γδ T Cells

Gamma delta (γδ) T cells are a population of unconventional T cells, expressing a TCR comprised of γ and δ chains, as opposed to the α and β chains of conventional T cells. The ability to produce IL-17A or IFNγ is largely predetermined in the thymus during development, alongside Vγ TCR chain expression, and these cells go on to seed tissues, particularly mucosal sites (3). γδ T cells develop in waves during development, with cells bearing a particular Vγ chain developing in the thymus in a pre-determined time window from which they seed specific tissues (4). γδ T cells have been suggested to operate in an ‘adaptate’ manner with both innate and adaptive features and their role is to ‘set the scene’ for full adaptive responses (4, 58). The innate functions of γδ T cells are evident by the fact that they are not restricted to recognition of antigens presented by MHC/CD1/MR1 (4), which is in contrast to conventional T cells. Indeed, there has been considerable debate regarding the importance and identity of TCR ligands for γδ T cells, although they are largely thought to be microbial or stress-associated molecules (58). γδ T cell subsets have been shown to play roles in repair *via* their production of cytokines, growth factors and antimicrobial peptides which will be discussed in the subsequent sections.

### IL-17-Producing γδ T Cells

A common feature of pro-repair γδ T cells is their type 17 polarization. In mouse skin, where their repair functions have been best characterized, γδ T cells comprise around 90% of all dermal IL-17 producers, with the major dermal γδ T cell population expressing Vγ4 (59) [Tonegawa nomenclature (60)]. Vγ4 T cells are able to infiltrate the epidermis on wounding, *via* a CCR6-CCL20 axis (61), with hair follicle (HF) epithelial cells also producing high levels of CCL20 (62). This suggests that in addition to migrating to the epidermis, IL-17 producing-γδ T cells may localize close to HF where they could facilitate stem cell mobilization *via* IL-17 production (26) (**Figure 2**).

CCR6 deficient mice display delayed healing associated with reduced recruitment of Vγ4 expressing γδ T cells and lack of pro-repair FGF2 at the wound site. However, these mice have increased IL-17 at day 5 post wounding (63) suggesting an alternative population of cells can produce IL-17, but do not compensate for the early repair functions carried out by γδ T cells.

Diabetic mice display impaired wound healing which is associated with reduced CCL20, IL-23 and IL-1β, reduced IL-17-producing γδ T cells and reduced CCR6 expression (64). This association again suggests a role for dermal γδ T in promoting repair, but the relative importance of these cells to healing is yet to be determined.

#### γδ T Cell-Derived IL-17

A major subset of γδ T cells are capable of producing copious amounts of IL-17. However, the role of Vγ4 T cell derived IL-17 remains controversial with conflicting reports of this impeding or enhancing wound healing. In support of a role promoting healing, IL-17 deficient mice have defective healing which can be restored by supplementing with IL-17 or transfer of IL-17 producing Vγ4 cells in a diabetic model (64). IL-17 neutralization has also been shown to delay skin healing, due to a reduction in the AMP, RegIIIγ (55) (**Figure 2**). Also, diabetic mouse wounds, with delayed healing have lower total IL-17, and reduced numbers of IL-17 producing γδ T cells (64). A recent study also identified reduced expression of IL-17/Type-17 related genes in human diabetic wound samples (57) demonstrating again an association between impaired healing and reduced IL-17. Conversely, Vγ4-derived IL-17 can delay healing by inhibiting insulin-like growth factor 1 (IGF-1) production by dendritic epidermal T cells (DETC) (61). It has therefore been suggested that there is a dose dependent role for IL-17 in wound healing, where low levels of IL-17 promote healing, but once a threshold is exceeded, IL-17 produced largely by dermal γδ T cells, triggers a positive feedback loop enhancing IL-1β and IL-23 production (61, 65). This results in disruption of growth factor release in the wound and further enhanced IL-17 production, impairing healing (61).

The requirement for a type-17 response for wound healing is surprising given that healing is commonly thought of as a type-2 inflammatory response. However, type-17 responses have been shown to be required for the promotion of subsequent type-2 responses in helminth infections, where significant tissue damage occurs and requires repair (66). More specifically, IL-17 is necessary for later IL-13 production (67) and the promotion of a later type-2 response may depend upon neutrophil mediated damage or IL-17-mediated suppression of IFNγ responses to relieve inhibition of type-2 responses (66). Indeed, this is illustrated in neonatal influenza infection, where a rapid γδ T cell IL-17A response is generated in the lung, which induces IL-33 in epithelial cells, leading to a pro-repair Areg response from ILC2 and Tregs (68) (**Figure 3**). Mice deficient in γδ T cells develop more severe disease associated with reduced weight gain and prolonged lung pathology on infection with influenza, which is due to impaired repair processes rather than an impaired anti-viral response. The IL-33/IL-17A repair axis may also operate in human infants, as nasal aspirates from children with mild influenza show positive correlations between IL-17A and IL-33 levels and also between IL-17A and Areg levels (68). In turn, higher IL-17A levels were associated with less severe disease, implying a protective pro-repair role for IL-17A (68).

IL-17 responses may co-operate with a variety of growth factors in promoting barrier regeneration. In oral mucosa, IL-17 producing γδ T cells also produce Areg which promotes homeostasis and plays a protective role in periodontitis (69). Areg, as discussed for ILCs, has roles in healing at other mucosal sites.

Therefore, in healthy healing γδ T cell IL-17 production appears to be an early response to wounding which allows and promotes a subsequent type 2 inflammatory and pro-healing response to occur.

#### Role of γδ T Cells in Tissue Regeneration

It is important to note that repair often does not involve true regeneration. This is especially obvious in skin scarring, where tissue is repaired but loses much of its strength, elasticity and appendages, such as hair follicles, sebaceous glands and sweat glands. A particularly interesting aspect of γδ T cell-mediated repair is its capacity to be both healing and regenerative, restoring all cell types and original features of the tissue. In humans, regeneration, such as wound-induced hair neogenesis (**Figure 2**) - the de-novo generation of hair follicles at the site of a repaired wound- is extremely rare, but regeneration can be observed in mouse injury models. This difference may well be due to resident dermal γδ T cells, as human dermis contains fewer γδ T cells, and those few present in human skin localize around dermal vasculature suggesting that they are circulating rather than tissue resident cells (70). Murine dermal γδ T cells are thought to promote regeneration as the regenerative mouse-like species of the genus *Acomys* have greater transcription of *Tcrd* in the wound edge in late stage healing than in *Mus.musculus* (71). Dermal γδ T cells are also the main FGF9 producers late in healing (70) (**Figure 2**) which promotes a positive feedback mechanism in dermal fibroblasts resulting in further FGF9 production triggering Wnt signaling in the dermis. As in development, Wnt signalling helps to promote hair neogenesis in wounds, which requires γδ T cells (70). It is likely that Wnt signaling also favors regenerative and scarless healing. A recent study described how imiquimod, which promotes a type 17 response in skin, enhanced skin regeneration in a murine model (71). Imiquimod activates nociceptor neurons and induces IL-23 expression in dermal dendritic cells which promotes regeneration functions in γδ T cells (71).

### Intraepithelial γδ T Cells

#### DETC

In addition to the dermis, γδ T cell subsets are also resident in the epidermis, a major example being dendritic epidermal T cells (DETCs) (**Figure 2**) which express an invariant Vγ5Vδ1 TCR (72). DETCs are named for their dendritic appearance with projections that survey the epidermis and detect stress ligands *via* a range of costimulatory receptors such as NKG2D, which is important in their wound healing activities (73). Despite the identification of these costimulatory receptors, the actual TCR ligands involved are currently unknown and may not always be required for cell activation (58, 72). DETCs have an important role in maintaining skin homeostasis by producing the growth factor, IGF-1, which reduces keratinocyte apoptosis at steady state.

DETCs do not exist in humans due to inactivating mutations of the critical DETC selection protein Skint-1 (72). However, populations which appear to fulfill similar roles are present in human skin such as Vδ1 cells which reside in the epidermis but lack the dendritic morphology of DETCs (72, 74). Epidermal resident γδ T cells have also been identified in other mammalian species such as cattle and macaques and the presence of γδ T cells with tissue support roles is a conserved feature as they are found in evolutionarily distant species such as jawless fish (72).

The most convincing evidence for DETC playing a role in healing comes from mice lacking DETCs, where wound healing defects are observed in the early post-wounding period. This is likely due to loss of growth factors such as IGF-1, KGFs (Keratinocyte growth factors) and FGF-10 (75, 76) (**Figure 2**). DETC can be activated by TCR signaling, leading to the rapid production of IGF-1, which supports keratinocyte survival (77). IGF-1, KGFs and FGF-10 produced by DETC also enhance epidermal proliferation (61, 65, 75, 78, 79) and DETC adoptive transfer can rescue the wound healing defect and restore growth factor levels in TCRδ deficient animals (61). This suggests a major role for DETC in healing (75), which αβ T cells are unable to fill despite populating the epidermis in their absence.

DETC are activated in wounding which is characterized by morphologic changes triggered by keratinocyte stress ligands (77, 80). This change in morphology requires expression of semaphorin CD100 by DETC, and upregulation of its receptor, plexin B2 by keratinocytes (80). The importance of CD100 in repair, was shown by CD100 deficient animals, which have an impaired wounding response (80).

In a tape-stripping model of epidermal injury, DETCs were found to be important IL-13 producers which promotes KC maturation and migration through the epidermal layers to restore barrier function (81). This has similarities to unconventional epidermal αβ T cells which have a joint type 17-type 2 profile and promote wound healing in mice (82). It is therefore possible that in human skin where DETC are absent, a comparable αβ T cell subset might fulfil similar roles to murine DETCs. Additionally, Vδ1 T cells in human epidermis produce IGF-1 which is upregulated in response to activation *via* CD3 signaling and promotes acute wound healing (74). Therefore, these human skin γδ T cells may fill a similar role to murine DETC, albeit as a much rarer cell population.

Diminished DETC function is seen in murine models of impaired healing such as aged and diabetic models. In diabetic mice there is a steady-state impairment in DETC function, due to chronic TNFα exposure (83) and diminished keratinocyte IL-15 production, which normally support DETC homeostasis (84). In aged mice, DETC activation is impaired due to wound-edge keratinocytes failing to upregulate Skint proteins (85). Human epidermal γδ T cells are also impaired in chronic wounds, producing less IGF-1 than acute wounds (74), and suggesting a similar role for epidermal γδ T cells in mice and humans.

#### Intestinal IELs

The intestinal epithelium is also host to intraepithelial γδ T cells, but at this site they express Vγ7 and, in contrast to DETCs, do not display a dendritic morphology (**Figure 1**). These intestinal IELs are predisposed to IFNγ production and are motile, allowing the monitoring of intestinal integrity (3). Similar to DETC, the pro-healing activity of intestinal γδ IELs appears to be dependent upon growth factor production.

In the DSS-induced colitis model, repair is delayed in the absence of γδ T cells, with transmural ulcers and impaired epithelial proliferation and re-epithelialisation (86). Protection from this pathology was linked to γδ IEL production of KGF (86) (**Figure 1**), and, as seen in skin DETC (80), these responses are promoted by γδ IEL expression of CD100 and epithelial expression of the ligand, plexin B2 (87). The importance of CD100 is demonstrated in CD100 deficient mice which have impaired gut repair and worsened pathology in response to DSS colitis with a reduction in IEL proliferation and KGF-1 production (87).

In addition to growth factors, intestinal IELs can produce antimicrobial peptides which contribute to repair. During DSS colitis, damage seen in γδ T cell-deficient animals is accompanied with enhanced bacterial translocation. This is likely due, at least in part, to a reduction in the anti-microbial peptide RegIIIγ (88), (**Figure 1**) which alongside its antimicrobial role can also promote healing (55, 89). Intestinal IELs also contribute to anti-microbial peptide production by producing angiogenin 4, which emphasizes the multi-faceted role of this cell type in barrier protection (90). This study also demonstrates IL-22 production by intestinal γδ IELs (90), potentially further aiding in gut barrier maintenance.

Intestinal γδ IELs can also act to strengthen tight junctions, in part, *via* promoting phosphorylation of the tight junction protein, occludin which results in a limiting of gut permeability (91).

To identify other mechanisms by which γδ IELs promote repair, transcriptional profiling of γδ IELs was performed in DSS-induced colitis. This demonstrated a mixed profile of increased signatures related to inflammatory cell recruitment, antimicrobial function and cytoprotection, with expression of chemokines, lysozyme and heat shock proteins as well as βig-h3 (also known as TGFBI) which is known to promote keratinocyte healing (88). Therefore, it is likely that there are additional mechanisms involved in the promotion of repair by γδ IELs which are yet to be fully characterized. Interestingly, unlike DETC, there is a clear intestinal γδ IEL population in humans (92, 93) which may, therefore carry out similar roles to murine equivalents however, human intestinal IEL are dominated by αβ T cells (94).

Across differing γδ T cell subsets and different tissue localizations, we see a commonality in IL-17 producing cells participating in tissue repair and an essential role for growth factor production. This type-17 healing link certainly warrants further investigation since pro-healing roles have not been reported for IFNγ-producing γδ T cells other than IEL. There is also an intriguing link between γδ T cell-mediated repair mechanisms and a more regenerative form of repair, which could provide insight into factors driving regeneration versus repair. While intraepithelial subsets do not possess an IL-17 producing capacity, the contribution of these to tissue repair may largely rest on their ability to produce a range of growth factors.

## Mucosal-Associated Invariant T Cells (MAIT)

MAIT, or Mucosal-associated invariant T cells, are unconventional T cells bearing a semi-invariant αβ TCR which recognizes molecules presented by the non-classical MHC molecule MR1 (2). MAIT are known to populate human mucosal tissues such as the gastrointestinal tract and lung, as well as being highly prevalent in peripheral blood and the liver (95, 96). In mice, these cells are present in a range of mucosal sites such as the small intestinal lamina propria and lung, as well as being present in liver, spleen and thymus, and these cells are particularly enriched in mouse skin (97).

MAIT play an important role in microbial surveillance *via* their MR1-restricted TCR. MR1 presents riboflavin (vitamin B2) synthesis intermediates to MAIT which acts as a non-self signal (**Figures 1**–**3**), since mammals cannot implement this synthetic pathway, enabling the detection of bacteria and fungi that synthesize riboflavin (2). On detection of these intermediates, MAIT promote anti-bacterial and anti-viral responses *via* a mixed type-1 type-17 response involving both IL-17 and IFNγ production (98). In humans a dual type-1/17 phenotype is displayed by individual MAIT cells (99), while in mice it appears that there are different types of MAIT cells capable of either type-17 or type-1 inflammation, with the type-17 MAIT being more common in mouse models (95). The presence of MAIT is critically dependent upon the microbiota, in particular, riboflavin synthesizing commensals, which provide metabolites for presentation *via* MR1 (97).

Alongside other ‘Unconventional T cells’ (including, iNKT and γδ T cells), MAIT cells are enriched within murine skin (97). The greatest number of skin MAIT cells are tissue-resident DN (CD4-, CD8-) MAIT cells, residing at the epidermal-dermal junction (**Figure 2**) (97). Skin MAIT cells require IL-23 for homeostasis and have enriched gene signatures for type-17 inflammation (RORγt, IL-23R, IL-17, IL-22, CCR6), relative to skin CD4^+^ T cells (97). These cells are also enriched for healing related genes such as *Lgals3* [Galectin 3 (100)] and *Sdc1*- (Syndecan – a proteoglycan with documented repair roles) (97, 101) (**Figure 2**). Skin colonization with the commensal *Staphylococcus epidermidis* causes TCR and IL-18-dependent expansion of the MAIT cell population, and upregulation of both proinflammatory and repair-related genes including *Ceacam1, Grn, Hmox1, Igf1, Pdgfa*, and pro-angiogenic genes such as angiogenin (97) (**Figure 2**). IGF-1 and PDGF are also produced by DETC to promote repair, so there may be similarities between the repair mechanisms of DETC and MAIT cells. The enhanced production of pro-angiogenic factors by MAIT is also interesting, as angiogenesis is an important component of healing.

Mouse MAIT cells promote healing as cutaneous application of MAIT cell ligand 5-OP-RU results in enhanced cutaneous healing (97). Conversely, mice lacking MAIT cells on a TCRδ deficient background have impaired healing, involving delayed re-epithelialisation (97). *In vitro* wound assays also demonstrate a role for MAIT cells in repair of colonic epithelium (37) (**Figure 1**). In addition, in steady state human colon, MAIT can be found closely associated with colonic epithelium, a highly appropriate location for these cells to undertake both surveillance and repair functions (37). MAIT cells have also been shown to limit diabetes development in the NOD (non-obese diabetic) mouse model by strengthening intestinal epithelial integrity *via* production of IL-17, IL-22 and promoting expression of mucin-2 and occludin (102). In this model the absence of MAIT cells increased intestinal permeability and was associated with bacterial translocation (102), again demonstrating a crucial role for MAIT cell maintenance of barrier integrity.

Akin to findings in the gut and skin, activation of murine MAIT cells by *in vivo* lung infection or *in vitro* stimulation enhances the expression of repair associated genes (103). In both scenarios MAIT respond to activation by producing mixed type-17 and type 1 cytokines IL-17A, IL-17F, IL-22, IFNγ and TNFα. They also express growth factors of the EGF family (such as amphiregulin and HBEGF), Platelet derived growth factor B (PDGFB) and VEGF family (VEGFA/B), and proteases such as furin and members of the MMP family (103) (**Figure 3**) which all contribute to repair. Therefore, similar to skin and gut, there is strong evidence of lung MAIT cells producing important pro-repair factors upon activation.

While TCR-independent activation of MAIT cells is possible, *via* IL-12 and IL-18, TCR stimulation is required to induce repair associated genes, suggesting MAIT cells may be particularly important in repair in response to infection or microbial incursion (37, 99). Although stimulation of MAIT with cytokines such as IL-12, IL-18, IL-15 and TL1A also upregulated some genes linked to repair such as furin, many other pro-repair genes were downregulated and proinflammatory genes such as IL-17F were upregulated (37, 99). This is suggestive of MAIT cells having a pro-repair phenotype aiding the maintenance of epithelial integrity in intact tissue when they are only exposed to TCR stimuli, however, when damage occurs the MAIT cells will receive both cytokine (such as IL-18) signals alongside TCR stimulation which will promote a mixed inflammatory/anti-microbial and repair phenotype. Interestingly, *in vitro* studies of human peripheral blood MAIT cells showed that TCR signaling promotes RORγt and IL-17 expression, while cytokine stimulation promotes Tbet expression (99), which could suggest similar type-17 and repair phenotypes in human as in mouse MAIT cells, with both responses being promoted by TCR signaling.

Many comparisons have been made between MAIT and H2-M3-restricted innate-like CD8 T cells known to promote skin healing. These cells are also commensal-specific and demonstrate a type-17 polarization with a poised transcriptional landscape favoring type 2, or ‘wound healing’ responses (82). Therefore, MAIT cells and these innate-like CD8 T cells may have similar functions. Further research should determine if the roles of these cells are redundant, or if there are repair functions specific to each cell type.

Therefore, multiple studies of human and murine MAIT cells from different sites all demonstrate a propensity toward a TCR-dependent repair program. However, one issue with studies to date is that the broad transcriptional analyses have not identified the most important mechanisms of repair utilized by MAIT. Indeed, it may be the case that these factors display some redundancy, however, identifying important mechanisms could highlight novel therapeutic targets and may inform whether targeting MAIT represents a potential therapeutic strategy in conditions where repair is insufficient.

## Discussion

### A Repair Convention Within Unconventional Lymphocytes

Unconventional lymphocytes such as ILCs, γδ T cells and MAIT cells contribute to tissue repair by employing common mechanisms, such as production of growth factors, IL-17, IL-22, Areg, antimicrobial peptide production and the regulation of myeloid cell activity. These similarities between the cells might reflect a requirement for similar repair responses to be initiated on damage with a variety of microbes present. Indeed, MAIT, IL-17-producing γδ T cells, and ILC3 are dependent on commensals for development and function, and in some cases, commensals are directly involved in their repair function, as seen for MAIT. Conversely, ILC2 also possess pro-healing activities but are not dependent on the microbiota (21), however, this may reflect a more delayed role in healing for ILC2, as they act during the late proliferative to remodeling phase, potentially in response to IL-33 released in response to type-17 inflammation (68).

The ability to produce growth factors such as Areg, IGF-1 and PDGF is shared across different unconventional lymphocyte populations, and appears to underlie much of these cells’ repair role. Cytokine production is also an important mechanism for unconventional lymphocytes in repair, providing an early source of cytokines such as IL-17, IL-13 and IL-5, which are otherwise mainly produced by adaptive immune cells. This could be especially important in ‘sterile’ injury or situations where there is no dominant infectious agent to target with an adaptive response. Indeed, MAIT cells, ILC and γδ T cells are all responsive to cytokines frequently released in damage such as IL-1β (ILC3, γδ T cells), IL-18 (MAIT, ILC2), IL-33 and IL-25 (ILC2) and so are well-placed to respond to tissue damage. Furthermore, these lymphocyte subsets can be activated solely *via* cytokine signaling enabling a rapid response to damage signals and changing tissue signals as the site repairs. Another important role early in repair for unconventional lymphocyte cytokine production is influencing myeloid cell recruitment and activity. Myeloid cells are often a major focus of repair studies, however determining how much of this activity is dependent upon early ILC activity would be of interest.

While ILC2 play an important role in barrier repair, this review also highlights a predominance of IL-17-producing cells such as γδ T Cells, MAIT and ILC3 in tissue and barrier repair. Indeed, in the γδ T cell field the role of IL-17 is well-studied with a suggestion that a moderate IL-17 response is important for healing, whereas extremes in IL-17A levels are detrimental (61, 65). IL-17 production is tightly linked to commensal presence, and the early IL-17 response may act to promote epithelial proliferation to rapidly reconstitute the barrier and thus prevent microbial invasion.

Given the many effector mechanisms and repair strategies employed by unconventional lymphocytes, a challenge will be identifying which population is the critical contributor. Once key meditators, such as Areg are identified, the use of conditional knockouts or cell transfer experiments of intact cells into mediator-deficient recipients will help determine which populations are sufficient for repair *via* a given mechanism. This will avoid the niche-shifting effect of genetic or antibody mediated depletion of unconventional lymphocyte populations.

Unconventional lymphocytes are poised to play critical role in barrier repair – they are well placed as often resident cells or cells that can be rapidly recruited with an ability to respond to damage signals and produce mediators that act on the epithelium to restore barrier function. There are differences in unconventional lymphocyte populations, so an important question is how this affects repair.

### Unconventional Lymphocytes and Human Healing

Although many of the detailed functional assays to date have exploited murine models it is important to note that human unconventional lymphocytes share features in common with murine equivalents. However, there do seem to be some differences in function and composition. For instance, DETC are a high frequency population in murine skin but are lacking in human skin, and overall MAIT cells are more frequent in human than in murine tissues (96, 97, 104), particularly in human skin where MAIT cells occupy a far greater share of the niche than γδ T cells (97, 105). Human MAIT cells may also take up some of the iNKT niche, a lymphocyte subset also over-represented in mice relative to humans (95), however, human MAIT are found in the intestine at low levels, comparable to that seen in blood (37, 96). Despite differences in relative frequencies, the repair signature of activated MAIT appears remarkably consistent (37) and suggests a repair role for these cells in both species. Furthermore, there is evidence for γδ T cells participating in human wound repair since they have been shown to produce IGF-1 (alongside αβ T cells) in acute wounds but not in chronic non healing wounds (74). Therefore, it is likely that in humans, unconventional lymphocytes play important roles in repair, but the relative contribution of individual subsets may differ from mice. This highlights an important need to utilize both human and murine studies in order to best establish the commonalities and differences in order to better understand barrier integrity and repair.

### Site Specific Healing

This review has discussed unconventional lymphocytes in lung, skin and gut and it is notable that there are many similarities in responses between tissues. While ILC, for example, display a high degree of tissue dependent gene signatures (21, 106, 107), their reported mechanisms for promoting barrier repair are remarkably similar across sites. We have also seen a common theme among ILC2 for responses to IL-33 resulting in Areg production, with IL-13 playing a myeloid or stem cell instructive role. Additionally, MAIT cells isolated from various tissues and across mouse and human display similar pro-repair signatures. Therefore, there may be core, conserved, programs of repair within these populations irrespective of site or species. The field is currently lacking mechanistic detail for the important mediators of repair, however, and while core mechanisms clearly exist, there may be further specialized pro-repair pathways which are activated in a more context dependent or site-specific manner.

### Regenerative Healing

Work describing regenerative healing in rodent species suggest an important role of IL-17 producing γδ T cells (71) although a direct role for IL-17 has not been shown for regeneration. Instead the regenerative role of γδ T cells may lie in their growth factor producing abilities. This is supported by imiquimod (IMQ)-induced skin regeneration which requires IL-17 producing γδ T cells (71) despite the presence of other type-17 cells, such as ILC3 (108) that are activated by IMQ and produce IL-17 (109, 110). This is therefore suggestive of IL-17-producing γδ T cells having a regenerative role that cannot be compensated for by 17 production from either ILC3, or MAIT. Therefore pro-regenerative capacity is not conferred by IL-17, but may be dependent on production of morphogenic growth factors such as FGF9 (70). This mechanism warrants a thorough investigation to allow the identification of the pro-regeneration factors and investigate the potential to utilize such discoveries as novel therapies.

### Future Directions and Perspectives

A specific limitation to increasing our understanding of the role of ILCs in repair, is the relative lack of specific ILC subset knockout models. Instead, studies to date largely relied upon comparisons between RAG KO and RAGxIL-2R KO or RAG KO anti-Thy1 treated animals which may overestimate the role of ILC by presenting their activity in the absence of T and B cells where ILC numbers are typically increased and appear hyperactivated (111). These approaches could also miss important ILC-T cell cross talk in barrier repair thus failing to accurately represent the role of ILCs in an otherwise immunocompetent host. Therefore, the development of additional *in vivo* tools to understand these cells will be invaluable in the future. Limitations in the study of unconventional lymphocytes will be aided with increased single cell techniques, as a large number of markers are often needed to successfully identify and phenotype these populations *in situ*. This is all the more important when we consider that many of these populations, particularly ILC and γδ T cells, are extremely tailored to their tissue site, so may have specific tissue adaptations. Furthermore, questions still remain about differences in cell populations between mice and humans, in particular regarding which populations are analogous between the species. A better understanding of this basic biology should be established to allow comparative work to progress.

Although it is clear that unconventional lymphocytes contribute to repair, currently little is known about the source of these cells in injury, specifically whether they proliferate *in situ* or are recruited from a distal site. Likewise, the fate of unconventional lymphocytes post injury, is unknown. Whether these populations retract by apoptosis, or retain any imprinted memory of the injury that could allow a more rapid or more tailored repair program during subsequent injury is currently undetermined, but warrants further investigation. Such knowledge could be invaluable in the treatment of diseases such as diabetes, in which there are reductions in unconventional lymphocytes which is associated with the development of chronic wounds. Determining mechanisms of restoring these cell types to the tissue could identify novel therapeutic targets which would greatly benefit the repair process.

While many studies have shown that unconventional lymphocytes promote tissue repair, mechanistic detail is still largely missing. Additionally, while skin is the main tissue in which repair is studied, there is a notable gap in the literature for mechanistic details of ILCs in repair and barrier maintenance. This may be due to the technical challenges in isolating ILC without functionally altering these cells in harsh tissue digestion protocols.

Another remaining gap is the identification of ligands that can trigger unconventional lymphocyte activation. For example, while it is recognized that γδ TCR ligands involved in barrier repair are stress-induced, some are yet to be defined, and the exact nature of these ligands is elusive. Significant efforts and progress are now being made in identifying γδ TCR ligands and this will likely give further insight into the repair roles of γδ T cells which will present opportunities to modulate their activity.

The more we understand about these unconventional lymphocytes, the more scope there is to promote the activity of unconventional lymphocytes therapeutically to promote tissue repair. Conceptually this may be more easily achievable for MAIT cells where the ligand, 5-OP-RU could be applied to the injury site and would provide the key TCR signal for pro-repair gene expression. Given the huge changes to mechanisms involved in the repair process at different time points, any therapeutic would require careful timing of dosing to ensure appropriate activity within the correct phase of the healing process. Given the shared repair mechanisms of a number of unconventional lymphocyte subsets, and the difficulties in specifically targeting subsets of these cells, it may be more beneficial to identify the crucial mediators of repair, such as amphiregulin which could then be targeted therapeutically, rather than focusing on the cell subtype involved.

The study of unconventional lymphocytes is a rapidly developing field. While this review has drawn together some of the roles they play in barrier sites, there is much still to be learnt and the potential for them in therapy could be an exciting avenue for future research.

## Author Contributions

JC conceived the article, JC and AS conducted the literature review. Manuscript was written and revised by JC, AS, and SC. All authors contributed to the article and approved the submitted version.

## Funding

This work was supported by a Wellcome Trust Sir Henry Dale Fellowship to AS (109375/Z/15/Z) and a Medical Research Council (MRC) DTP studentship to JC.

## Conflict of Interest

The authors declare that the research was conducted in the absence of any commercial or financial relationships that could be construed as a potential conflict of interest.

## References

[1] Baarsma HA Skronska-Wasek W Mutze K Ciolek F Wagner DE John-Schuster G. Noncanonical WNT-5A signaling impairs endogenous lung repair in COPD. J Exp Med (2017) 214:143–63. 10.1084/jem.20160675 PMC5206496 27979969

[2] Kjer-Nielsen L Patel O Corbett AJ Le Nours J Meehan B Liu L. MR1 presents microbial vitamin B metabolites to MAIT cells. Nature (2012) 491:717–23. 10.1038/nature11605 23051753

[3] Ribot JC Lopes N Silva-Santos B. γδ T cells in tissue physiology and surveillance. Nat Rev Immunol (2020) 10:221–32. 10.1038/s41577-020-00452-4 33057185

[4] Hayday AC. γδ T Cell Update: Adaptate Orchestrators of Immune Surveillance. J Immunol (2019) 203:311–20. 10.4049/jimmunol.1800934 31285310

[5] Vivier E Artis D Colonna M Diefenbach A Di Santo JP Eberl G. Innate Lymphoid Cells: 10 Years On. Cell (2018) 174:1054–66. 10.1016/j.cell.2018.07.017 30142344

[6] Melo-Gonzalez F Hepworth MR. Functional and phenotypic heterogeneity of group 3 innate lymphoid cells. Immunology (2017) 150:265–75. 10.1111/imm.12697 PMC5290240 27935637

[7] Spits H Bernink JH Lanier L. NK cells and type 1 innate lymphoid cells: Partners in host defense. Nat Immunol (2016) 17:758–64. 10.1038/ni.3482 27328005

[8] Weizman O Adams NM Schuster IS Krishna C Pritykin Y Lau C. ILC1 Confer Early Host Protection at Initial Sites of Viral Infection. Cell (2017) 171:795–808.e12. 10.1016/j.cell.2017.09.052 29056343 PMC5687850

[9] McGinty JW von Moltke J. A three course menu for ILC and bystander T cell activation. Curr Opin Immunol (2020) 62:15–21. 10.1016/j.coi.2019.11.005 31830683 PMC7067623

[10] Halim TYF Krauß RH Sun AC Takei F. Lung Natural Helper Cells Are a Critical Source of Th2 Cell-Type Cytokines in Protease Allergen-Induced Airway Inflammation. Immunity (2012) 36:451–63. 10.1016/j.immuni.2011.12.020 22425247

[11] Robinette ML Fuchs A Cortez VS Lee JS Wang Y Durum SK. Transcriptional programs define molecular characteristics of innate lymphoid cell classes and subsets. Nat Immunol (2015) 16:306–17. 10.1038/ni.3094 PMC4372143 25621825

[12] Bernink JH Peters CP Munneke M Te Velde AA Meijer SL Weijer K. Human type 1 innate lymphoid cells accumulate in inflamed mucosal tissues. Nat Immunol (2013) 14:221–9. 10.1038/ni.2534 23334791

[13] Jowett GM Norman MDA Yu TTL Rosell Arévalo P Hoogland D Lust ST. ILC1 drive intestinal epithelial and matrix remodelling. Nat Mater (2020) 20:250–9. 10.1038/s41563-020-0783-8 PMC7611574 32895507

[14] Li Z Hodgkinson T Gothard EJ Boroumand S Lamb R Cummins I. Epidermal Notch1 recruits RORγ + group 3 innate lymphoid cells to orchestrate normal skin repair. Nat Commun (2016) 7:11394. 10.1038/ncomms11394 27099134 PMC4844683

[15] Xu J Zanvit P Hu L Tseng PY Liu N Wang F. The Cytokine TGF-β Induces Interleukin-31 Expression from Dermal Dendritic Cells to Activate Sensory Neurons and Stimulate Wound Itching. Immunity (2020) 53:371–83. 10.1016/j.immuni.2020.06.023 PMC7362873 32673566

[16] Rak GD Osborne LC Siracusa MC Kim BS Wang K Bayat A. IL-33-Dependent Group 2 Innate Lymphoid Cells Promote Cutaneous Wound Healing. J Invest Dermatol (2016) 136:487–96. 10.1038/JID.2015.406 PMC4731037 26802241

[17] Monticelli LA Sonnenberg GF Abt MC Alenghat T Ziegler CGK Doering TA. Innate lymphoid cells promote lung-tissue homeostasis after infection with influenza virus. Nat Immunol (2011) 12:1045–54. 10.1038/ni.2131 PMC3320042 21946417

[18] Huang Y Guo L Qiu J Chen X Hu-Li J Siebenlist U. IL-25-responsive, lineage-negative KLRG1hi cells are multipotential ‘inflammatory’ type 2 innate lymphoid cells. Nat Immunol (2015) 16:161–9. 10.1038/ni.3078 PMC4297567 25531830

[19] Turner J-E Morrison PJ Wilhelm C Wilson M Ahlfors H Renauld J-C. IL-9–mediated survival of type 2 innate lymphoid cells promotes damage control in helminth-induced lung inflammation. J Exp Med (2013) 210:2951–65. 10.1084/jem.20130071 PMC3865473 24249111

[20] Dagher R Copenhaver AM Besnard V Berlin A Hamidi F Maret M. IL-33-ST2 axis regulates myeloid cell differentiation and activation enabling effective club cell regeneration. Nat Commun (2020) 11:4786–810. 10.1038/s41467-020-18466-w PMC7508874 32963227

[21] Ricardo-Gonzalez RR Van Dyken SJ Schneider C Lee J Nussbaum JC Liang H-EE. Tissue signals imprint ILC2 identity with anticipatory function. Nat Immunol (2018) 19:1093–9. 10.1038/s41590-018-0201-4 PMC6202223 30201992

[22] Gieseck RL Wilson MS Wynn TA. Type 2 immunity in tissue repair and fibrosis. Nat Rev Immunol (2018) 18:62–76. 10.1038/nri.2017.90 28853443

[23] Zhu P Zhu X Wu J He L Lu T Wang Y. IL-13 secreted by ILC2s promotes the self-renewal of intestinal stem cells through circular RNA circPan3. Nat Immunol (2019) 20:183–94. 10.1038/s41590-018-0297-6 30643264

[24] Dahlgren MW Jones SW Cautivo KM Dubinin A Ortiz-Carpena JF Farhat S. Adventitial Stromal Cells Define Group 2 Innate Lymphoid Cell Tissue Niches. Immunity (2019) 50:707–22.e6. 10.1016/j.immuni.2019.02.002 30824323 PMC6553479

[25] Garcin CL Ansell DM. The battle of the bulge: re-evaluating hair follicle stem cells in wound repair. Exp Dermatol (2017) 26:101–4. 10.1111/exd.13184 27574799

[26] Chen X Cai G Liu C Zhao J Gu C Wu L. IL-17R-EGFR axis links wound healing to tumorigenesis in Lrig1+ stem cells. J Exp Med (2019) 216:195–214. 10.1084/jem.20171849 30578323 PMC6314525

[27] Mathur AN Zirak B Boothby IC Tan M Cohen JN Mauro TM. Treg-Cell Control of a CXCL5-IL-17 Inflammatory Axis Promotes Hair-Follicle-Stem-Cell Differentiation During Skin-Barrier Repair. Immunity (2019) 50:655–67.e4. 10.1016/j.immuni.2019.02.013 30893588 PMC6507428

[28] Johnson AMF Costanzo A Gareau MG Armando AM Quehenberger O Jameson JM. High fat diet causes depletion of intestinal eosinophils associated with intestinal permeability. PloS One (2015) 10:1–15. 10.1371/journal.pone.0122195 PMC4383570 25837594

[29] Wu D Molofsky AB Liang H-E Ricardo-Gonzalez RR Jouihan HA Bando JK. Eosinophils Sustain Adipose Alternatively Activated Macrophages Associated with Glucose Homeostasis. Science (80- ) (2011) 332:243–7. 10.1126/science.1201475 PMC3144160 21436399

[30] Toor IS Rückerl D Mair I Ainsworth R Meloni M Spiroski AM. Eosinophil Deficiency Promotes Aberrant Repair and Adverse Remodeling Following Acute Myocardial Infarction. JACC Basic to Transl Sci (2020) 5:665–81. 10.1016/j.jacbts.2020.05.005 PMC7393409 32760855

[31] Zaiss DMW Gause WC Osborne LC Artis D. Emerging functions of amphiregulin in orchestrating immunity, inflammation, and tissue repair. Immunity (2015) 42:216–26. 10.1016/j.immuni.2015.01.020 PMC4792035 25692699

[32] Monticelli LA Osborne LC Noti M Tran SV Zaiss DMW Artis D. IL-33 promotes an innate immune pathway of intestinal tissue protection dependent on amphiregulin-EGFR interactions. Proc Natl Acad Sci U S A (2015) 112:10762–7. 10.1073/pnas.1509070112 PMC4553775 26243875

[33] Cho HS Reboldi A Hall JA Berg LJ. The Tec kinase ITK is essential for ILC2 survival and epithelial integrity in the intestine. Nat Commun (2019) 10:784–96. 10.1038/s41467-019-08699-9 PMC6377622 30770814

[34] Kobayashi T Voisin B Kim DY Kennedy EA Jo J-HH Shih H-YY. Homeostatic Control of Sebaceous Glands by Innate Lymphoid Cells Regulates Commensal Bacteria Equilibrium. Cell (2019) 176:982–97.e16. 10.1016/j.cell.2018.12.031 30712873 PMC6532063

[35] Salimi M Barlow JL Saunders SP Xue L Gutowska-Owsiak D Wang X. A role for IL-25 and IL-33–driven type-2 innate lymphoid cells in atopic dermatitis. J Exp Med (2013) 210:2939–50. 10.1084/jem.20130351 PMC3865470 24323357

[36] Zeis P Lian M Fan X Herman JS Hernandez DC Gentek R. In Situ Maturation and Tissue Adaptation of Type 2 Innate Lymphoid Cell Progenitors. Immunity (2020) 53:775–92. 10.1016/j.immuni.2020.09.002 PMC7611573 33002412

[37] Leng T Akther HD Hackstein C-P Powell K King T Friedrich M. TCR and Inflammatory Signals Tune Human MAIT Cells to Exert Specific Tissue Repair and Effector Functions. Cell Rep (2019) 28:3077–91.e5. 10.1016/j.celrep.2019.08.050 31533032 PMC6899450

[38] Zhang Y Zhou M Wei H Zhou H He J Lu Y. Furin promotes epithelial-mesenchymal transition in pancreatic cancer cells via Hippo-YAP pathway. Int J Oncol (2017) 50:1352–62. 10.3892/ijo.2017.3896 28259973

[39] Candi E Schmidt R Melino G. The cornified envelope: A model of cell death in the skin. Nat Rev Mol Cell Biol (2005) 6:328–40. 10.1038/nrm1619 15803139

[40] Ghaedi M Shen ZY Orangi M Martinez-Gonzalez I Wei L Lu X. Single-cell analysis of RORα tracer mouse lung reveals ILC progenitors and effector ILC2 subsets. J Exp Med (2020) 217:1–19. 10.1084/jem.20182293 PMC7062532 31816636

[41] Aparicio-Domingo P Romera-Hernandez M Karrich JJ Cornelissen F Papazian N Lindenbergh-Kortleve DJ. Type 3 innate lymphoid cells maintain intestinal epithelial stem cells after tissue damage. J Exp Med (2015) 212:1783–91. 10.1084/jem.20150318 PMC4612094 26392223

[42] Castro-Dopico T Fleming A Dennison TW Ferdinand JR Harcourt K Stewart BJ. GM-CSF Calibrates Macrophage Defense and Wound Healing Programs during Intestinal Infection and Inflammation. Cell Rep (2020) 32:107857–72. 10.1016/j.celrep.2020.107857 PMC7351110 32640223

[43] Mann A Breuhahn K Schirmacher P Blessing M. Keratinocyte-derived granulocyte-macrophage colony stimulating factor accelerates wound healing: Stimulation of keratinocyte proliferation, granulation tissue formation, and vascularization. J Invest Dermatol (2001) 117:1382–90. 10.1046/j.0022-202x.2001.01600.x 11886498

[44] Fang Y Gong SJ Xu YH Hambly BD Bao S. Impaired cutaneous wound healing in granulocyte/macrophage colony-stimulating factor knockout mice. Br J Dermatol (2007) 157:458–65. 10.1111/j.1365-2133.2007.07979.x 17553038

[45] Sonnenberg GF Fouser LA Artis D. Border patrol: Regulation of immunity, inflammation and tissue homeostasis at barrier surfaces by IL-22. Nat Immunol (2011) 12:383–90. 10.1038/ni.2025 21502992

[46] Romera-Hernández M Aparicio-Domingo P Papazian N Karrich JJ Cornelissen F Hoogenboezem RM. Yap1-Driven Intestinal Repair Is Controlled by Group 3 Innate Lymphoid Cells. Cell Rep (2020) 30:37–45.e3. 10.1016/j.celrep.2019.11.115 31914395

[47] Duffin R OConnor RA Crittenden S Forster T Yu C Zheng X. Prostaglandin E2 constrains systemic inflammation through an innate lymphoid cell-IL-22 axis. Science (80- ) (2016) 351:1333–8. 10.1126/science.aad9903 PMC4841390 26989254

[48] Chun E Lavoie S Fonseca-Pereira D Bae S Michaud M Hoveyda HR. Metabolite-Sensing Receptor Ffar2 Regulates Colonic Group 3 Innate Lymphoid Cells and Gut Immunity. Immunity (2019) 51:871–84.e6. 10.1016/j.immuni.2019.09.014 31628054 PMC6901086

[49] Gronke K Hernández PP Zimmermann J Klose CSN Kofoed-Branzk M Guendel F. Interleukin-22 protects intestinal stem cells against genotoxic stress. Nature (2019) 566:249–53. 10.1038/s41586-019-0899-7 PMC6420091 30700914

[50] Malhotra N Yoon J Leyva-Castillo JM Galand C Archer N Miller LS. IL-22 derived from γδ T cells restricts Staphylococcus aureus infection of mechanically injured skin. J Allergy Clin Immunol (2016) 138:1098–107. 10.1016/j.jaci.2016.07.001 PMC5056816 27543072

[51] Avitabile S Odorisio T Madonna S Eyerich S Guerra L Eyerich K. Interleukin-22 Promotes Wound Repair in Diabetes by Improving Keratinocyte Pro-Healing Functions. J Invest Dermatol (2015) 135:2862–70. 10.1038/jid.2015.278 26168231

[52] Kolumam G Wu X Lee WP Hackney JA Zavala-Solorio J Gandham V. IL-22R ligands IL-20, IL-22, and IL-24 promote wound healing in diabetic db/db mice. PloS One (2017) 12:1–20. 10.1371/journal.pone.0170639 PMC5268431 28125663

[53] Liang SC Tan X-Y Luxenberg DP Karim R Dunussi-Joannopoulos K Collins M. Interleukin (IL)-22 and IL-17 are coexpressed by Th17 cells and cooperatively enhance expression of antimicrobial peptides. J Exp Med (2006) 203:2271–9. 10.1084/jem.20061308 PMC2118116 16982811

[54] McGeachy MJ Cua DJ Gaffen SL. The IL-17 Family of Cytokines in Health and Disease. Immunity (2019) 50:892–906. 10.1016/j.immuni.2019.03.021 30995505 PMC6474359

[55] Lai Y Li D Li C Muehleisen B Radek KA Park HJ. The Antimicrobial Protein REG3A Regulates Keratinocyte Proliferation and Differentiation after Skin Injury. Immunity (2012) 37:74–84. 10.1016/j.immuni.2012.04.010 22727489 PMC3828049

[56] Wu L Chen X Zhao J Martin B Zepp JA Ko JS. A novel IL-17 signaling pathway controlling keratinocyte proliferation and tumorigenesis via the TRAF4-ERK5 axis. J Exp Med (2015) 212:1571–87. 10.1084/jem.20150204 PMC4577838 26347473

[57] Sawaya AP Stone RC Brooks SR Pastar I Jozic I Hasneen K. Deregulated immune cell recruitment orchestrated by FOXM1 impairs human diabetic wound healing. Nat Commun (2020) 11:4678. 10.1038/s41467-020-18276-0 32938916 PMC7495445

[58] Vermijlen D Gatti D Kouzeli A Rus T Eberl M. γδ T cell responses: How many ligands will it take till we know? Semin Cell Dev Biol (2018) 84:75–86. 10.1016/j.semcdb.2017.10.009 29402644

[59] Cai Y Shen X Ding C Qi C Li K Li X. Pivotal Role of Dermal IL-17-Producing γδ T Cells in Skin Inflammation. Immunity (2011) 35:596–610. 10.1016/j.immuni.2011.08.001 21982596 PMC3205267

[60] Heilig JS Tonegawa S. Diversity of murine gamma genes and expression in fetal and adult T lymphocytes. Nature (1986) 322:836–40. 10.1038/322836a0 2943999

[61] Li Y Wang Y Zhou L Liu M Liang G Yan R. Vγ4 T cells inhibit the pro-healing functions of dendritic epidermal T cells to delay skin wound closure through IL-17A. Front Immunol (2018) 9:240. 10.3389/fimmu.2018.00240 29483920 PMC5816340

[62] Kabashima K Honda T Ginhoux F Egawa G. The immunological anatomy of the skin. Nat Rev Immunol (2019) 19:19–30. 10.1038/s41577-018-0084-5 30429578

[63] Anderson LS Yu S Rivara KR Reynolds MB Hernandez AA Wu X. CCR6+ γδ T Cells Home to Skin Wounds and Restore Normal Wound Healing in CCR6-Deficient Mice. J Invest Dermatol (2019) 139:2061–4.e2. 10.1016/j.jid.2019.02.032 PMC6708754 30935975

[64] Liu Z Xu Y Zhang X Liang G Chen L Xie J. Defects in dermal Vγ4 γ δ T cells result in delayed wound healing in diabetic mice. Am J Transl Res (2016) 8:2667–80. PMC4931161 27398150

[65] Li Y Wu J Luo G He W. Functions of Vγ4 T Cells and Dendritic Epidermal T Cells on Skin Wound Healing. Front Immunol (2018) 9:1099. 10.3389/fimmu.2018.01099 29915573 PMC5994537

[66] Allen JE Sutherland TE Rückerl D. IL-17 and neutrophils: Unexpected players in the type 2 immune response. Curr Opin Immunol (2015) 34:99–106. 10.1016/j.coi.2015.03.001 25794823

[67] Sutherland TE Logan N Rückerl D Humbles AA Allan SM Papayannopoulos V. Chitinase-like proteins promote IL-17-mediated neutrophilia in a tradeoff between nematode killing and host damage. Nat Immunol (2014) 15:1116–25. 10.1038/ni.3023 PMC4338525 25326751

[68] Guo X zhi J Dash P Crawford JC Allen EK Zamora AE Boyd DF. Lung γδ T Cells Mediate Protective Responses during Neonatal Influenza Infection that Are Associated with Type 2 Immunity. Immunity (2018) 49:531–44.e6. 10.1016/j.immuni.2018.07.011 30170813 PMC6345262

[69] Krishnan S Prise IE Wemyss K Schenck LP Bridgeman HM McClure FA. Amphiregulin-producing γδ T cells are vital for safeguarding oral barrier immune homeostasis. Proc Natl Acad Sci (2018) 115:10738–43. 10.1073/pnas.1802320115 PMC6196490 30279177

[70] Gay D Kwon O Zhang Z Spata M Plikus MV Holler PD. Fgf9 from dermal γδ T cells induces hair follicle neogenesis after wounding. Nat Med (2013) 19:916–23. 10.1038/nm.3181 PMC4054871 23727932

[71] Wei JJ Kim HS Spencer CA Brennan-Crispi D Zheng Y Johnson NM. Activation of TRPA1 nociceptor promotes systemic adult mammalian skin regeneration. Sci Immunol (2020) 5:1–9. 10.1126/SCIIMMUNOL.ABA5683 PMC7703669 32859683

[72] Sutoh Y Mohamed RH Kasahara M. Origin and Evolution of Dendritic Epidermal T Cells. Front Immunol (2018) 9:1059. 10.3389/fimmu.2018.01059 29868019 PMC5960712

[73] Yoshida S Mohamed RH Kajikawa M Koizumi J Tanaka M Fugo K. Involvement of an NKG2D Ligand H60c in Epidermal Dendritic T Cell-Mediated Wound Repair. J Immunol (2012) 188:3972–9. 10.4049/jimmunol.1102886 22403443

[74] Toulon A Breton L Taylor KR Tenenhaus M Bhavsar D Lanigan C. A role for human skin-resident T cells in wound healing. J Exp Med (2009) 206:743–50. 10.1084/jem.20081787 PMC2715110 19307328

[75] Jameson J Ugarte K Chen N Yachi P Fuchs E Boismenu R. A role for skin γδ T cells in wound repair. Science (80- ) (2002) 296:747–9. 10.1126/science.1069639 11976459

[76] Havran WL Jameson JM. Epidermal T Cells and Wound Healing. J Immunol (2010) 184:5423–8. 10.4049/jimmunol.0902733 PMC2944652 20483798

[77] Komori HK Witherden DA Kelly R Sendaydiego K Jameson JM Teyton L. Cutting edge: dendritic epidermal γδ T cell ligands are rapidly and locally expressed by keratinocytes following cutaneous wounding. J Immunol (2012) 188:2972–6. 10.4049/jimmunol.1100887 PMC3311739 22393149

[78] Jameson JM Cauvi G Witherden DA Havran WL. A Keratinocyte-Responsive γδ TCR Is Necessary for Dendritic Epidermal T Cell Activation by Damaged Keratinocytes and Maintenance in the Epidermis. J Immunol (2004) 172:3573–9. 10.4049/jimmunol.172.6.3573 15004158

[79] Sharp LL Jameson JM Cauvi G Havran WL. Dendritic epidermal T cells regulate skin homeostasis through local production of insulin-like growth factor 1. Nat Immunol (2005) 6:73–9. 10.1038/ni1152 15592472

[80] Witherden DA Watanabe M Garijo O Rieder SE Sarkisyan G Cronin SJF. The CD100 Receptor Interacts with Its Plexin B2 Ligand to Regulate Epidermal γδ T Cell Function. Immunity (2012) 37:314–25. 10.1016/j.immuni.2012.05.026 PMC3430606 22902232

[81] Dalessandri T Crawford G Hayes M Castro Seoane R Strid J. IL-13 from intraepithelial lymphocytes regulates tissue homeostasis and protects against carcinogenesis in the skin. Nat Commun (2016) 7:12080–92. 10.1038/ncomms12080 PMC4931319 27357235

[82] Harrison OJ Linehan JL Shih H Bouladoux N Han S Smelkinson M. Commensal-specific T cell plasticity promotes rapid tissue adaptation to injury. Science (80- ) (2019) 363:eaat6280. 10.1126/science.aat6280 PMC7304459 30523076

[83] Taylor KR Mills RE Costanzo AE Jameson JM. γδ T Cells Are Reduced and Rendered Unresponsive by Hyperglycemia and Chronic TNFα in Mouse Models of Obesity and Metabolic Disease. PLoS One (2010) 5:e11422. 10.1371/journal.pone.0011422 20625397 PMC2896399

[84] Liu Z Liang G Gui L Li Y Liu M Bai Y. Weakened IL-15 Production and Impaired mTOR Activation Alter Dendritic Epidermal T Cell Homeostasis in Diabetic Mice. Sci Rep (2017) 7:1–10. 10.1038/s41598-017-05950-5 28729536 PMC5519720

[85] Keyes BE Liu S Asare A Naik S Levorse J Polak L. Impaired Epidermal to Dendritic T Cell Signaling Slows Wound Repair in Aged Skin. Cell (2016) 167:1323–38.e14. 10.1016/j.cell.2016.10.052 27863246 PMC5364946

[86] Chen Y Chou K Fuchs E Havran WL Boismenu R. Protection of the intestinal mucosa by intraepithelial γδ T cells. Proc Natl Acad Sci U S A (2002) 99:14338–43. 10.1073/pnas.212290499 PMC137885 12376619

[87] Meehan TF Witherden DA Kim CH Sendaydiego K Ye I Garijo O. Protection against colitis by CD100-dependent modulation of intraepithelial γδ T lymphocyte function. Mucosal Immunol (2014) 7:134–42. 10.1038/mi.2013.32 PMC3795871 23695512

[88] Ismail AS Behrendt CL Hooper LV. Reciprocal Interactions between Commensal Bacteria and γδ Intraepithelial Lymphocytes during Mucosal Injury. J Immunol (2009) 182:3047–54. 10.4049/jimmunol.0802705 PMC2763635 19234201

[89] Zhao D Kim YH Jeong S Greenson JK Chaudhry MS Hoepting M. Survival signal REG3α prevents crypt apoptosis to control acute gastrointestinal graft-versus-host disease. J Clin Invest (2018) 128:4970–9. 10.1172/JCI99261 PMC6205404 30106382

[90] Walker CR Hautefort I Dalton JE Overweg K Egan CE Bongaerts RJ. Intestinal intraepithelial lymphocyte-enterocyte crosstalk regulates production of bactericidal angiogenin 4 by paneth cells upon microbial challenge. PLoS One (2013) 8:1–16. 10.1371/journal.pone.0084553 PMC3866140 24358364

[91] Dalton JE Cruickshank SM Egan CE Mears R Newton DJ Andrew EM. Intraepithelial γδ+ Lymphocytes Maintain the Integrity of Intestinal Epithelial Tight Junctions in Response to Infection. Gastroenterology (2006) 131:818–29. 10.1053/j.gastro.2006.06.003 16952551

[92] Di Marco Barros R Roberts NA Dart RJ Vantourout P Jandke A Nussbaumer O. Epithelia Use Butyrophilin-like Molecules to Shape Organ-Specific γδ T Cell Compartments. Cell (2016) 167:203–18.e17. 10.1016/j.cell.2016.08.030 27641500 PMC5037318

[93] Melandri D Zlatareva I Chaleil RAG Dart RJ Chancellor A Nussbaumer O. The γδTCR combines innate immunity with adaptive immunity by utilizing spatially distinct regions for agonist selection and antigen responsiveness. Nat Immunol (2018) 19:1352. 10.1038/s41590-018-0253-5 30420626 PMC6874498

[94] Vandereyken M James OJ Swamy M. Mechanisms of activation of innate-like intraepithelial T lymphocytes. Mucosal Immunol (2020) 13:721–31. 10.1038/s41385-020-0294-6 PMC7434593 32415229

[95] Provine NM Klenerman P. MAIT Cells in Health and Disease. Annu Rev Immunol (2020) 38:203–28. 10.1146/annurev-immunol-080719-015428 31986071

[96] Dusseaux M Martin E Serriari N Péguillet I Premel V Louis D. Human MAIT cells are xenobiotic-resistant, tissue-targeted, CD161 hi IL-17-secreting T cells. Blood (2011) 117:1250–9. 10.1182/blood-2010-08-303339 21084709

[97] Constantinides MG Link VM Tamoutounour S Wong AC Perez-Chaparro PJ Han S-J. MAIT cells are imprinted by the microbiota in early life and promote tissue repair. Science (2019) 366:6624–37. 10.1126/science.aax6624 PMC7603427 31649166

[98] Hinks TSC Zhang XW. MAIT Cell Activation and Functions. Front Immunol (2020) 11:1014. 10.3389/fimmu.2020.01014 32536923 PMC7267072

[99] Lamichhane R Schneider M de la Harpe SM Harrop TWR Hannaway RF Dearden PK. TCR- or Cytokine-Activated CD8+ Mucosal-Associated Invariant T Cells Are Rapid Polyfunctional Effectors That Can Coordinate Immune Responses. Cell Rep (2019) 28:3061–76.e5. 10.1016/j.celrep.2019.08.054 31533031

[100] McLeod K Walker JT Hamilton DW. Galectin-3 regulation of wound healing and fibrotic processes: insights for chronic skin wound therapeutics. J Cell Commun Signal (2018) 12:281–7. 10.1007/s12079-018-0453-7 PMC5842207 29372416

[101] Stepp MA Pal-Ghosh S Tadvalkar G Pajoohesh-Ganji A. Syndecan-1 and Its Expanding List of Contacts. Adv Wound Care (2015) 4:235–49. 10.1089/wound.2014.0555 PMC4397989 25945286

[102] Rouxel O Da Silva J Beaudoin L Nel I Tard C Cagninacci L. Cytotoxic and regulatory roles of mucosal-associated invariant T cells in type 1 diabetes. Nat Immunol (2017) 18:1321–31. 10.1038/ni.3854 PMC6025738 28991267

[103] Hinks TSC Marchi E Jabeen M Olshansky M Kurioka A Pediongco TJ. Activation and In Vivo Evolution of the MAIT Cell Transcriptome in Mice and Humans Reveals Tissue Repair Functionality. Cell Rep (2019) 28:3249–62.e5. 10.1016/j.celrep.2019.07.039 31533045 PMC6859474

[104] Rahimpour A Koay HF Enders A Clanchy R Eckle SBG Meehan B. Identification of phenotypically and functionally heterogeneous mouse mucosal-associated invariant T cells using MR1 tetramers. J Exp Med (2015) 212:1095–108. 10.1084/jem.20142110 PMC4493408 26101265

[105] Suwanpradid J Holcomb ZE MacLeod AS. Emerging Skin T-Cell Functions in Response to Environmental Insults. J Invest Dermatol (2017) 137:288–94. 10.1016/j.jid.2016.08.013 PMC5552043 27784595

[106] Mazzurana L Czarnewski P Jonsson V Wigge L Ringnér M Williams TC. Tissue-specific transcriptional imprinting and heterogeneity in human innate lymphoid cells revealed by full-length single-cell RNA-sequencing. Cell Res (2021) 1–15. 10.1038/s41422-020-00445-x 33420427 PMC8089104

[107] Meininger I Carrasco A Rao A Soini T Kokkinou E Mjösberg J. Tissue-Specific Features of Innate Lymphoid Cells. Trends Immunol (2020) 41:902–17. 10.1016/j.it.2020.08.009 32917510

[108] Pantelyushin S Haak S Ingold B Kulig P Heppner FL Navarini AA. Rorγt + innate lymphocytes and γδ T cells initiate psoriasiform plaque formation in mice. J Clin Invest (2012) 122:2252–6. 10.1172/JCI61862 PMC3366412 22546855

[109] Sandrock I Reinhardt A Ravens S Binz C Wilharm A Martins J. Genetic models reveal origin, persistence and nonredundant functions of IL-17-producing γδ T cells. J Exp Med (2018) 215:3006–18. 10.1084/jem.20181439 PMC6279411 30455268

[110] Polese B Zhang H Thurairajah B King IL. Innate Lymphocytes in Psoriasis. Front Immunol (2020) 11:242. 10.3389/fimmu.2020.00242 32153574 PMC7047158

[111] Bando JK Colonna M. Innate lymphoid cell function in the context of adaptive immunity. Nat Immunol (2016) 17:783–9. 10.1038/ni.3484 PMC5156404 27328008

